# Multi-stimuli-responsive pectin-coated dendritic mesoporous silica nanoparticles with Eugenol as a sustained release nanocarrier for the control of tomato bacterial wilt

**DOI:** 10.1186/s12951-025-03239-8

**Published:** 2025-03-08

**Authors:** Xueping Guo, Huiyan Li, Zhihao Li, Ziqi Cui, Guangming Ma, Aisha Khalfan Nassor, Yi Guan, Xiaohong Pan

**Affiliations:** 1 State Key Laboratory of Agricultural and Forestry Biosecurity & Key Lab of Biopesticide and Chemical Biology, Ministry of Education & Ministerial and Provincial Joint Innovation Centre for Safety Production of Cross-Strait Crops, College of Plant Protection, Fujian Agriculture and Forestry University, Fuzhou, Fujian 350002 P. R. China; 2 Fujian Key Laboratory of Marine Enzyme Engineering, College of Biological Science and Engineering, Fuzhou University, Fuzhou, Fujian 350116 P.R. China

**Keywords:** Controlled release, Eugenol, Dendritic mesoporous silica nanoparticles, Tomato bacterial wilt

## Abstract

**Background:**

Environmentally responsive nanoscale biocide delivery system enhances smart, regulated, and synergistic biocide application with precise biocide release. In this study, pectin-modified dendritic mesoporous silica nanoparticles (DMSNs) was used as a carrier to successfully construct a microenvironment-responsive (pH, temperature and enzyme) eugenol nano-biocide delivery system for the control of *Ralstonia solanacearum* infection.

**Results:**

The results showed that the specific surface area, pore size and surface activity of DMSNs significantly influence the biocide loading of eugenol, and the biocide loading capability was up to 72.50%. Eu@DMSNs/Pec had significant pH and pectinase stimulating effects, with varying release amounts under different temperature conditions. Compared with eugenol alone, Eu@DMSNs/Pec significantly enhanced the efficacy of eugenol. DMSNs assisted eugenol to induce peroxidation damage, produce ROS (•O_2_^−^, •OH and ^1^O_2_), achieve synergistic antibacterial effects, and had better rain erosion resistance and foliar retention rate based on pectin wettability and adhesion. Eu@DMSNs/Pec-FITC showed demonstrated efficient transport characteristics in tomato roots, stems and leaves, which enhanced the control effect on tomato bacterial wilt. In addition, Eu@DMSNs/Pec exert minimal influence on tomato seed germination and root growth, and have low toxicity to non-target organisms such as earthworms. Therefore, Eu@DMSNs/Pec environment-responsive nano-controlled release nanocarrier can effectively achieve accurate biocide release and reduce biocide dosage.

**Conclusion:**

This work not only provides a pectin-modified DMSNs-based eugenol nanoscale biocide delivery system in response to specific environmental conditions of *R. solanacearum* infection but also elucidates the eugenol biocide loading, selective release ability and antibacterial mechanism of the system.

**Supplementary Information:**

The online version contains supplementary material available at 10.1186/s12951-025-03239-8.

## Graphical Abstract

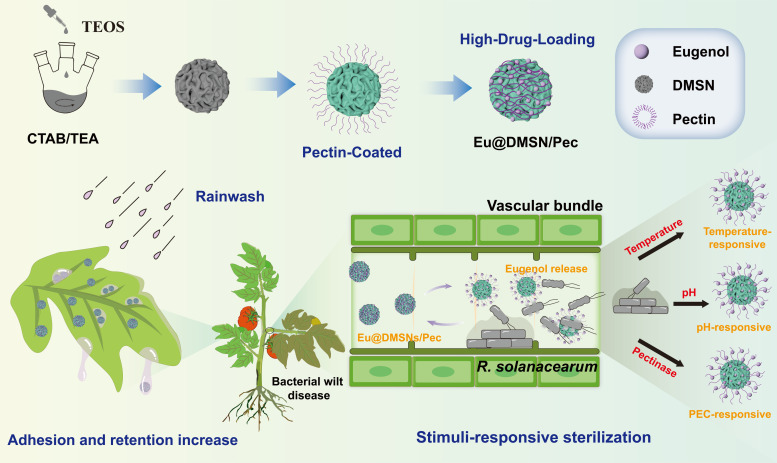

## Background

*Ralstonia solanacearum* is the pathogen of a global epidemic soil-borne disease - bacterial wilt, infects key crops like tomatoes, potatoes, and peppers, causing wilt and significant agricultural losses. Despite chemicals being the primary disease control method, they raise concerns about environmental impact, health, and food safety due to residues and resistance [1]. Thus, natural agricultural agrochemicals, known for their efficiency, low toxicity, and eco-friendliness, are emerging as a promising research focus with broad application potential [2]. Eugenol (Eu), a renewable monomer extracted from plants and the primary active component of clove essential oil, possesses a variety of biological activities including antimicrobial, antiviral, anti-fungal, anti-inflammatory, and antioxidant properties [3]. It has been widely used in various fields such as cosmetology, medicine, and pharmacology for a long time [4]. Wang et al. prepared Pectin/eugenol (Pe/Eu) composite film by casting method and obtained packaging materials with antibacterial and antioxidant properties [5]. Sun et al. discovered that eugenol boosts tomato resistance to yellow leaf curl virus, possibly by promoting nitric oxide and salicylic acid synthesis in tomatoes [6]. These eugenol characteristics make it show potential application value in the control of *R. solanacearum*.

However, as a plant essential oil, its volatility leads to its low persistence in the environment [7, 8], which affects its long-term effect as a germicide. Moreover, in the current research on biocide development, water-based, granulation and slow controlled release have become the mainstream biocide formulation development [9, 10]. When eugenol is formulated into a water-based biocide, its susceptibility to oxidation and hydrolysis increases, resulting in a decrease in the content of its active ingredients, thereby limiting the overall effectiveness and shelf life of the formulation [3].

In recent years, biocide-controlled release encapsulation technology has received extensive attention [9]. For example, researchers prepared pH-responsive MSNs-Chitosan@Prochloraz nanoparticles to reduce Prochloraz toxicity and optimize release performance [11]. Sharma et al. [12] developed a dual-stimulus-responsive halloysite nanotubes-polydopamine agricultural carrier for controlling bacterial soft rot, and also developed a triple intelligent environmental protection nanocarrier for pepper anthracnose prevention and control to reduce pesticide leaching and soil pollution [13]. Liang et al. [14] prepared pH-responsive ZnO@ZIF-8 nanospheres to release alkaloids on demand and synergistically and efficiently control bacterial wilt.

Meanwhile, researchers have adopted a various methods to improve the stability of eugenol in aqueous formulation [15, 16]. By encapsulating the active ingredients in nanocarriers, the dispersibility and stability of biocide can be significantly improved, the target deposition and dose transfer can be promoted, the loss and degradation can be reduced, and the dosage and frequency of biocide application can be reduced [17]. Shah et al. combined eugenol with the conjugates of whey protein isolate and maltodextrin to form a transparent nano-dispersion system, and the antibacterial effect in milk was more effective than free eugenol [18]. Yan et al. combined marine with star polycation (SPc) to form a complex, which reduced its particle size to nanoscale and significantly improved its insecticidal activity and persistence [19]. Chen et al. grafted eugenol and carvacrol on chitosan nanoparticles. The prepared nanoparticles have antioxidant and antibacterial properties, less cytotoxicity than pure essential oil [20]. Among these, dendritic mesoporous silica nanoparticles (DMSNs), with their large surface area, and ease of surface modification, have been explored as an effective carrier [21]. An additional advantage of DMSNs over other inorganic NPs is their relatively superior safety profile [22]. A typical example is that the FDA has approved colloidal silica for use as a glidant in the production of tablets [23]. Also, the widely used food additive E511 consists of amorphous silica NPs with a diameter of 100 nm [23]. Loading biocide into DMSNs can improve the absorption and transport behavior of biocide to plants, thereby improving its utilization efficiency, reducing the potential risks to non-target organisms and the environment, and achieving a safer and more eco-friendly disease control effect [24].

While encapsulation techniques enhance the stability and delivery of biocides, the controlled release of these active ingredients under specific environmental conditions is crucial for effective pathogen control. Developing controlled-release systems that respond to environmental changes is significantly important for improving the efficacy of biocides in preventing and treating *R. solanacearum*. It was noted that *R. solanacearum* can grow in a wide range of pH, but its pathogenicity and interaction with plants may be affected by pH. Studies have shown that *R. solanacearum* is more suitable for growth under neutral or weakly acidic pH conditions [25]. Moreover, temperature changes also can regulate the expression of genes related to the interaction between *R. solanacearum* and plants, and the optimal infection temperature is generally about 28℃ [26]. Eugenol is more stable under this condition and is suitable as an environmentally friendly biocide [27]. In addition, during the infection process, *R. solanacearum* can produce important pathogenic factors including cell wall degrading enzymes, which are essential for the colonization of *R. solanacearum* plants [28]. Pectin is the main component of plant cell wall, which is easily degraded by cell wall degrading enzymes, making it a response factor for targeted drug delivery system during the infection of *R. solanacearum* [29]. Moreover, studies have shown that the high content of pectin in the leaf’s cell wall facilitated the transfer of nanoparticles [30]. Therefore, modification of nanocarriers with pectin may improve the efficiency of biocide delivery. Based on these physiological characteristics, we proposed constructing a multi-responsive eugenol-loaded nanosystem. The purpose of this system is to release biocide on demand by targeting the microenvironment of *R. solanacearum* infection, which can intelligently prevent and control bacterial wilt while reducing the use of biocide.

**Scheme 1:**
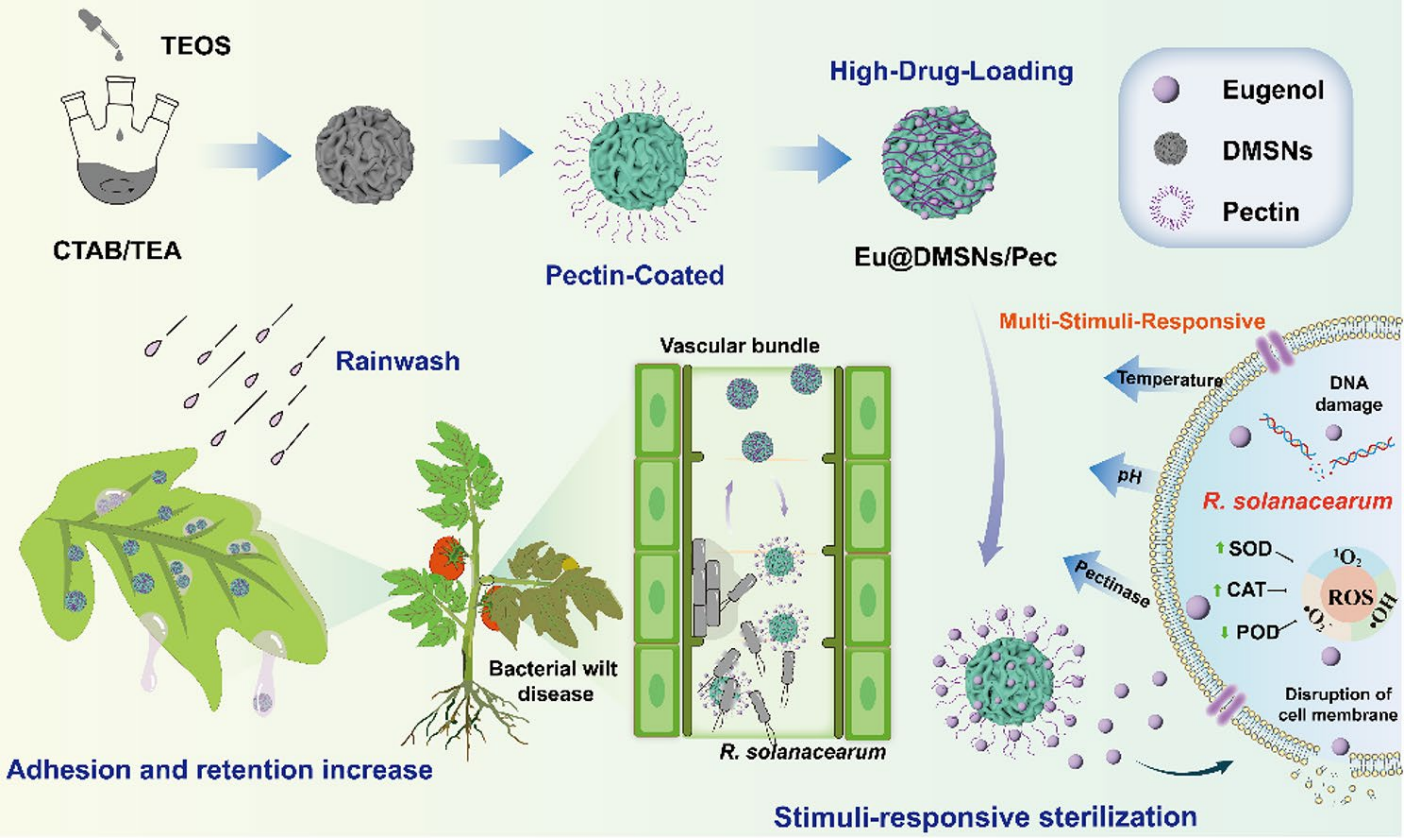
Preparation of Eu@DMSNs/Pec and their applications in smart control of tomato bacterial wilt. (CTAB: Cetyltrimethylammonium bromide, TEA: Triethanolamine, TEOS: Tetraethyl orthosilicate)

This study encompasses the development of highly loaded biocide delivery system for the release of hydrophobic eugenol based on a polymer carrier of DMSNs and pectin. The release performance, in vivo, in vitro biological activity and antibacterial mechanism against *R. solanacearum*, tomato leaf retention and distribution, tomato plant absorption and transport of the biocide-loaded nanosystem were systematically studied (as illustrated in Scheme 1). Its safety against non-target organisms was proved, providing a new application for sustainable nano-biocide nanocarrier as potential alternatives to traditional chemical biocides.

## Methods

### Materials

Triethylamine (TEA, ≥ 85%, Macklin), Cetyltrimethylammonium bromide (CTAB, 98%, Macklin), Sodium salicylate (NaSal, ≥ 99.5%, Macklin), Tetraethyl orthosilicate (TEOS, >99%, Macklin), 3-aminopropyltrimethoxysilane (APTES, 99%, Macklin), N, N-dimethylformamide (DMF, 99%, Macklin). 1-(3-dimethylaminopropyl)-3-ethylcarbodiimide (EDC, 98%, Solarbio), N-hydroxysuccinimide (NHS, 98%, Aladdin), Eugenol (Eu, 99%, Macklin).

### Dendritic mesoporous silica (DMSNs) preparation

The synthesis of dendritic mesoporous silica refers to the method of Yang et al. [31] 0.068 g TEA was dissolved in pure water (25 mL) and stirred in a water bath at 80 ℃ for 1 h. Then 380 mg CTAB and 168 mg NaSal were added to continue stirring for 1 h. Finally 4 mL TEOS was added to continue stirring for 2 h to obtain the reaction solution. The reaction solution was centrifuged at 4℃ and 11,000 r/min for 10 min to collect the precipitate [31]. The product was washed with pure water and anhydrous ethanol. The specific steps were as follows: the reaction mixture was centrifuged, washed with a large amount of pure water to neutral (pH = 7.0), and then washed with anhydrous ethanol three times. After freeze-drying, the precipitate was calcined at 550 °C for 4 h to obtain a delicate white powder, which was dendritic mesoporous silica. During the synthesis process, the appropriate stirring speed can effectively control the agglomeration rate of silicon species. The DMSNs was synthesized at 150, 200, 300 r/min, respectively, so that the silicon source was uniformly dispersed in the reaction system to avoid the formation of amorphous silica caused by excessive agglomeration [21].

### Preparation of Eu@DMSNs/Pec

**Fig. 1:**
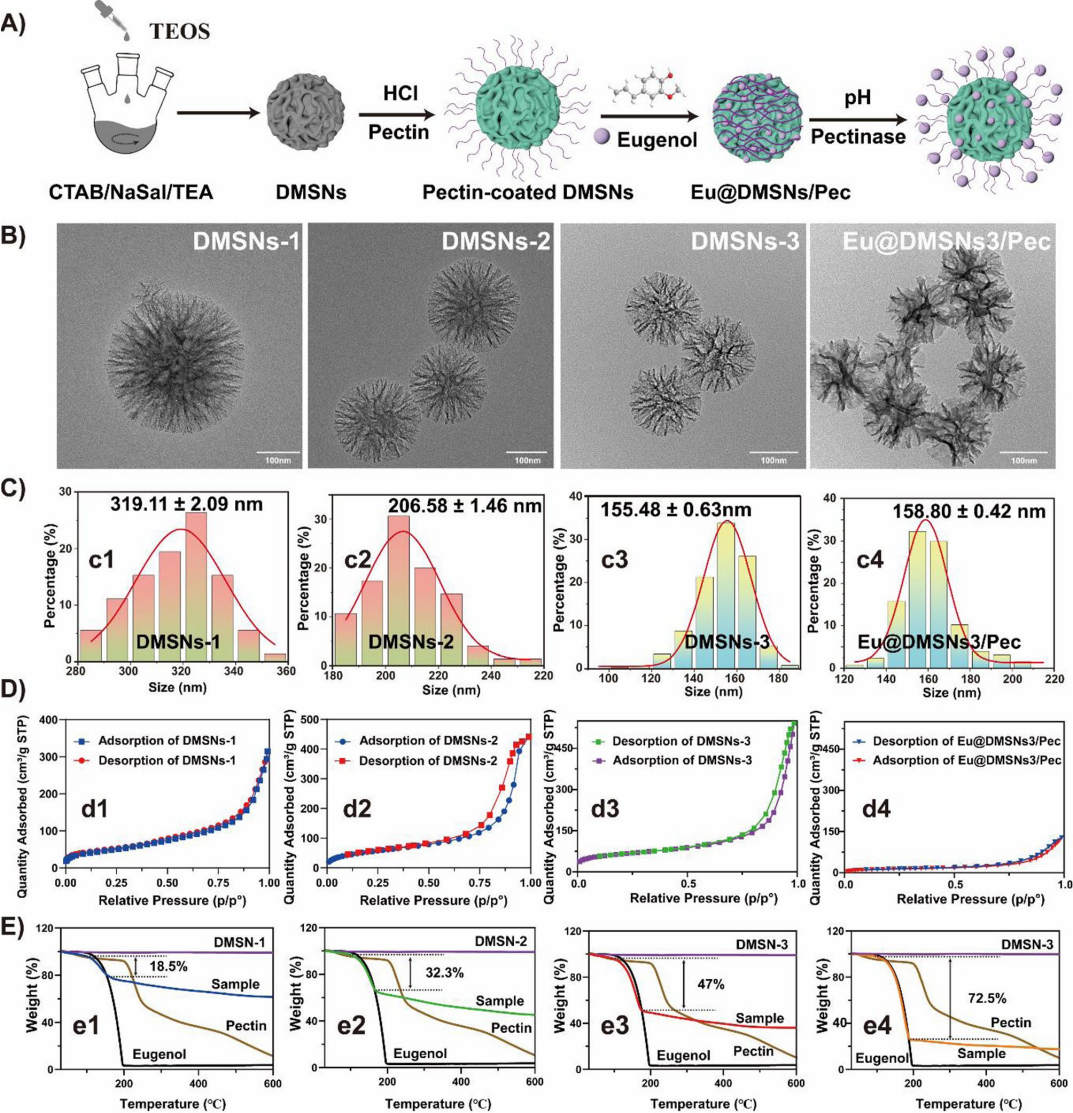
(**A**) Schematic illustration of the preparation of the Eu@DMSNs/Pec. (**B**) TEM images of DMSNs-1, DMSNs-2, DMSNs-3 and Eu@DMSN3/Pec. (**C**) Particle size distribution of (c1) DMSNs-1, (c2) DMSNs-2, (c3) DMSNs-3 and (c4) Eu@DMSNs3/Pec. (**D**) N_2_ adsorption/desorption isotherms curve of (d1) DMSNs-1, (d2) DMSNs-2, (d3) DMSNs-3 and (d4) Eu@DMSNs3/Pec (DMSNs3 was pretreated by HCl). (**E**) Thermogravimetric curve of (e1) Eu@DMSNs-1/Pec, (e2) Eu@DMSNs-2/Pec, (e3) Eu@DMSNs-3/Pec and (e4) Eu@DMSNs3/Pec (DMSNs3 was pretreated by HCl)

The process of pectin conjugation and eugenol loading is shown in Fig. 1A. The surface hydroxyl groups of DMSNs are activated by hydrochloric acid treatment, the amino groups are cross-linked by APTES in DMF solution, the pectin is grafted on the surface by amide reaction, and then eugenol is loaded. Finally, the precipitation is collected by centrifugation and freeze-dried to obtain the product Eu@DMSNs/Pec. See Supporting Information for detailed experimental methods and reagent information.

### Characterization of nanoparticles

The characterization data and analysis of the morphology, composition, structure and properties of the synthesized DMSNs and Eu@DMSNs/Pec, including but not limited to transmission electron microscopy (TEM), scanning electron microscopy (SEM), X-ray diffraction (XRD), Fourier transform infrared spectroscopy (FTIR), thermogravimetric analysis (TGA), etc. Please see Supporting Information.

### In vitro biocide release study

The release process of eugenol from Eu@DMSNs/Pec in different pH (4.0, 6.8 and 9.6), different pH with pectinase (pH 4.0, 6.8 and 9.6) and different temperature with pectinase (pH 6.8, 20 ℃, 25 ℃, 30 ℃, 35 ℃ and 40 ℃) was studied by dialysis method. The release medium was deionized water solution. The sample (10 mg) was dispersed in 10 mL release medium and placed in a dialysis bag. The dialysis bag was sealed and added to the beaker to supplement the release medium to 500 mL. The beaker was placed on a multi-joint magnetic stirrer, and the rotor speed was set to 200 r/min during the release process. 2 mL samples were regularly extracted from the release medium and the same volume of fresh release medium was supplemented. Please see Supporting Information for more detailed detection methods.

$$\:Cumulative\left(\%\right)={\sum\:}_{t=0}^{t}\frac{{M}_{t}}{{M}_{0}}\times\:100$$

Where M_t_ is the cumulative amount of eugenol released to each sampling time point, t is the time of the release of Eu@DMSNs/Pec and M_0_ is the initial weight of the eugenol-loaded in the nano/microcapsules.

The release kinetics of eugenol from Eu@DMSNs/Pec was analyzed using zero-order, first-order, Higuchi, and Ritger-Peppas models as follows:

Zero-order model: $\:\frac{{M}_{t}}{{M}_{\infty\:}}=kt$

First-order model: $\:\frac{{M}_{t}}{{M}_{\infty\:}}=1-{e}^{-kt}$

Ritger Peppas model: $\:\frac{{M}_{t}}{{M}_{\infty\:}}=k{t}^{n}$

Where *M*_*t*_ is the amount of Eugenol released at time *t, M*_*∞*_ is the maximal amount of the released Eugenol at infinite time, *k* is the rate constant of the biocide, and *n* is the diffusion exponent.

### Antibacterial experiment

The strains of *R. solanacearum* were cultured by the Key Lab of Biopesticide and Chemical Biology of Fujian Agriculture and Forestry University. The inhibitory effects of free eugenol and Eu@DMSNs/Pec at different concentrations (100, 200, 500 and 1000 mg/L) on the growth of *R. solanacearum* were determined by LB plate coating method. The LB liquid medium containing different concentrations of biocides was prepared, and the bacteria were cultured in a constant temperature oscillation incubator at 250 r/min for 12 h. After that, the bacterial solution was diluted to 10^5^ and spread on the plate. After 12 h of culture, the number of colonies was observed to calculate the bacteriostatic rate, and each treatment was repeated three times.

$$\:Inhibition\:\left(\%\right)=\left(1-\frac{\text{c}\text{o}\text{l}\text{o}\text{n}\text{i}\text{e}\text{s}\:\text{o}\text{f}\:\text{e}\text{x}\text{p}\text{e}\text{r}\text{i}\text{m}\text{e}\text{n}\text{t}\text{a}\text{l}\:\text{g}\text{r}\text{o}\text{u}\text{p}}{\text{c}\text{o}\text{l}\text{o}\text{n}\text{i}\text{e}\text{s}\:\text{o}\text{f}\:\text{c}\text{o}\text{n}\text{t}\text{r}\text{o}\text{l}\:\text{g}\text{r}\text{o}\text{u}\text{p}}\right)\times\:100\%$$

The treated bacteria were washed with sterile water and stained with SYTO^®^9 and propidium iodide (PI) dye. After incubation at room temperature in dark for 30 min, the cells were observed under a confocal laser scanning microscope (CLSM). In addition, SEM and TEM were used to observe the effects of different treatments on the morphological structure and biofilm of *R. solanacearum*. The preparation and analysis of confocal samples are detailed in Supporting Information.

### Reactive oxygen species (ROS) production and DNA damage

The activities of superoxide dismutase and peroxidase were detected by nitrogen blue tetrazolium (NBT) method and guaiacol method, and the activity of catalase was detected by detecting the ability of different treated samples to decompose H_2_O_2_ [32]. The types of reactive oxygen species produced by different treatments were detected on an electron paramagnetic resonance (EPR) instrument. •OH and •O_2_^−^ radicals were captured with 5,5-dimethyl-1-pyridine N-oxide (DMPO, Sigma-Aldrich), ^1^O_2_ was captured with 2,2,6,6-tetramethylpiperidine (TEMP, Sigma-Aldrich). DCFH-DA dye was used for staining, and ROS induced by Rosup treatment was used as a positive control. After incubation at 37℃ for 20 min (upside down every 5 min), the production of ROS was observed under a laser confocal microscope. The total DNA of *R. solanacearum* was extracted, and the DNA damage was detected by agarose gel electrophoresis (gel concentration of 2%) with YeaRed (YEASEN, China) as the staining agent.

### Foliar adhesion and retention

In order to measure the wettability and spread ability of the suspension on tomato leaves, tomato leaves were cut off and attached to a glass slide. Eugenol and Eu@DMSNs/Pec were configured into 100 mg/L aqueous solution, and the contact angle was continuously recorded by an optical contact angle measuring instrument (Biolin, Theta Lite, Finland) for 10 s. Tomato leaves were sprayed with 500 mg/L eugenol and Eu@DMSNs/Pec aqueous solution, and the number of leaves in each treatment was the same. Atomizer (SeeSa, SX-MD1EB, China) was used to simulate rainfall after 6 h, and the leaves were washed 1 - 2 times with 8 mm rainfall. The distribution of eugenol and Eu@DMSNs/Pec on tomato leaves before and after simulated rainfall was observed under SEM. Then the leaves were soaked with anhydrous ethanol to dissolve the residual eugenol on the leaves. The eugenol contents on the leaf surface under different treatments was determined by an ultraviolet spectrophotometer, and the eugenol residue per unit area was calculated. Eugenol standard curve: The ethanol solution of 10, 20, 30, 60, 80 and 100 mg/L eugenol was accurately configured with a volumetric flask, and the absorbance OD value at 280 nm was determined to make a standard curve.

### In vitro antibacterial activity

The control effect of the biocide-loading system and free eugenol on tomato bacterial wilt was determined on tomato leaves. Two-month-old healthy tomato plants with good growth conditions were selected, and different concentrations of eugenol and Eu@DMSNs/Pec aqueous solution (50, 100 and 200 mg/L) were sprayed on tomato leaves. After 3 days [33] of culture in a 25 ℃, 70% moderate artificial climate box, the leaves were inoculated with *R. solanacearum* (about 2.5 × 10^8^ CFU/mL). After inoculation, the leaves were cultured in the incubator for 3 days to observe the incidence of the leaves. The calculation method for the plant leaf disease index and disease index was implemented according to Tab. S1. Deionized water treatment was used as a blank control, and each treatment was repeated three times.

### The uptake and translocation

To investigate uptake and translocation of Eu@DMSNs/Pec in tomato plants during the synthesis process, referring to the research method of Zhao et al. [24]. 20 mg APTES-functionalized DMSNs-NH_2_ was dispersed in 10 mL absolute ethanol, 1 mg FITC was added, and the two were stirred at room temperature for 24 h under 550 r/min to cross-link. Then the product was washed three times with ethanol and deionized water and suspended in PBS. The reaction was continued with the activated pectin solution according to the previous experimental method. After grafting pectin, eugenol was loaded to obtain Eu@DMSNs/Pec-FITC with green fluorescence. Healthy tomato seedlings were selected, their roots were cleaned with deionized water before being immersed in a 200 mg/L Eu@DMSNs/Pec-FITC solution after 24 h of hydroponic culture. Plant tissues were then fixed and sliced into 0.2 mm thickness. These sections were carefully placed on slides, smoothed with tweezers, and excess water was absorbed using filter paper. The distribution of Eu@DMSNs/Pec-FITC in the leaves, stems, and roots was observed using CLSM.

### Biosafety assessment

#### Safety evaluation of tomato seed germination and growth

Eugenol and Eu@DMSNs/Pec were dissolved in pure water to prepare treatment solutions with eugenol concentrations of 50, 100, 200, and 500 mg/L. Sterilized toilet paper was placed in each petri dish and added 5 mL of sterile was added, 9 sterilized tomato seeds were placed, with three replicates for each treatment. DMSNs and pure water were used as blank controls. Incubation was carried out in a light incubator (25 °C, light/dark times: 13 h/11 h, 75% humidity) [30]. The seed germination and root lengths were measured after 5 days of incubation.

#### Safety evaluation to earthworm

Eugenol and Eu@DMSNs/Pec were dissolved and mixed with the soil in a basin to which the calculated deionized water was added, thus maintaining a moisture content of 30%. 30 earthworms in each group, and each treatment repeated three groups. Then, the earthworms were placed in a light incubator with appropriate temperature and humidity levels. The number of earthworm deaths was recorded at 14 days in the experiment (earthworms were considered dead if they did not respond to a touch on the tail) [34].

#### Safety evaluation to Escherichia coli and Bacillus subtilis

Antibacterial activity was evaluated by growing the bacteria on the solid agar plate containing different concentrations of eugenol and Eu@DMSNs/Pec. Briefly, the *E. coli* and *B. subtilis* cultures were harvested during the logarithmic phase and washed at least three times with phosphate buffer saline (PBS, pH = 7.0) by centrifugation at 8000 *g* for 5 min. After that, the bacterial solution was diluted to 10^5^ and spread on the LB liquid medium containing different concentrations of biocides. After 12 h of culture, the number of colonies was observed to calculate the bacteriostatic rate and EC_50_, and each treatment was repeated three times.

#### Safety evaluation to adult zebrafish

Zebrafish used to detect the safety of Eu@DMSNs/Pec to aquatic organisms. Adult zebrafish acclimated for more than 7 days (27 ℃, 14/10 h day/night) under laboratory conditions were used as experimental subjects, 20 zebrafish in each group, and each treatment was repeated three times. They were exposed to free eugenol and Eu@DMSNs/Pec aqueous solution (0.1, 1 and 10 mg/L), respectively, and treated with water as a blank control. The number of dead zebrafish was recorded at 24 h, 72 h and 96 h, and the dead zebrafish was quickly removed.

### Statistical analysis

Origin software (Origin Pro 2024b, OriginLab Corp., USA) was used to plot and curve the particle size distribution and release performance. GraphPad Prism 8.0 (GraphPad Software, Inc., USA) was used to analyze the zeta potential, bactericidal activity, enzyme activity and other data. The results are presented as the mean ± standard deviation. SPSS software (IBM SPSS Statistics Version 21.0 for Windows, IBM Corp., USA) was used to analyze the significance. Significance levels were set at *p* < 0.05, with asterisks (*) representing significant differences between groups and double asterisks (**) indicating highly significant differences when *p* < 0.01. The labels a, b, and c on each graph represent the statistical differences between different groups. (*P* < 0.05).

## Result and discussion

### Synthesis and characterization of Eu@DMSNs/Pec

The synthesis process is shown in Fig. 1A. After amination modification of DMSNs in DMF solution, pectin is grafted onto the surface of DMSNs by amide reaction in deionized water solution. The process of loading eugenol was carried out in anhydrous ethanol. Pectin molecular chains tend to be flexible and random in solution, forming weak cross-linking and extensive entanglement structures [35]. However, its poor solubility in solvents (anhydrous ethanol) allows strong and dense intermolecular interactions to form a strong network structure. After mixing with eugenol in the solvent, the two rapidly and spontaneously formed a stable complex Eu@DMSNs/Pec.

The particles size of DMSNs synthesized at a rotation speed of 150, 200 and 300 r/min were measured to be 319.11 ± 2.09 nm (Fig. 1c1), 206.58 ± 1.46 nm (Fig. 1c2), and 155.48 ± 0.63 nm (Fig. 1c3), respectively. With the increased stirring rate, the particle size of DMSNs decreased (Fig. 1B). Rapid stirring helps to reduce the concentration of silica surfactant micelles, thereby inhibiting the growth of DMSNs and contributing to the acquisition of small-sized DMSNs [36]. The physical adsorption curve (Fig. 1D) shows that compared with DMSNs-1 (Fig. 1d1), DMSNs-2 (Fig. 1d2) and DMSNs-3 (Fig. 1d3) have H4-type hysteresis loops, indicating that their pore size distribution is wider and the mesoporous pore size is larger [37]. DMSNs with different particle sizes showed different loading effects (Fig. 1E). It was found that DMSNs with smaller particle size had larger specific surface area and pore volume (Table 1). The biocide loading capability after grafting pectin increased from 18.5% (Fig. 1e1) to 32.3% (Fig. 1e2) and 47% (Fig. 1e3). This may be because DMSNs with larger specific surface area and pore volume can crosslink and fix more pectin on the surface. The decrease of particle size and the increase of specific surface area increase the surface charge density of DMSNs, which leads to the increase of the absolute value of zeta potential [38] (Fig. S1 A). Through the adhesion characteristics of pectin and electrostatic adsorption, eugenol is fixed on the surface or near the surface of the material to achieve higher biocide loading capability. Accordingly, we used concentrated hydrochloric acid (HCl) to activate the surface of DMSNs. HCl can react with the silicon hydroxyl group (Si-OH) on the surface of mesoporous silicon to form silicon chloride (Si-Cl) and water [39]. In this process, the introduction of amino and thiol groups can increase the surface active site grafting amino (-NH_2_) [40], and then cross-linked to fix more pectin, so that the biocide loading capability of eugenol is as high as 72.5% in the final products (Fig. 1e4).

**Table 1:** Comparison of BET surface area, pore volume, pore size, and biocide loading capability of nanoparticles before and after biocide loading

Sample	BET (m²/g)	Pore Volume (cm³/g)	Pore Size (nm)	biocide Loading (%)
DMSNs1	136.84	0.49	11.07	—
Eu@DMSNs1/Pec	63.41	0.39	8.98	18.50
DMSNs2	203.02	0.68	13.44	—
Eu@DMSNs2/Pec	66.40	0.41	11.25	32.30
DMSNs3	237.24	0.84	14.09	—
Eu@DMSNs3/Pec	48.98	0.20	8.16	47.00

**Fig. 2:**
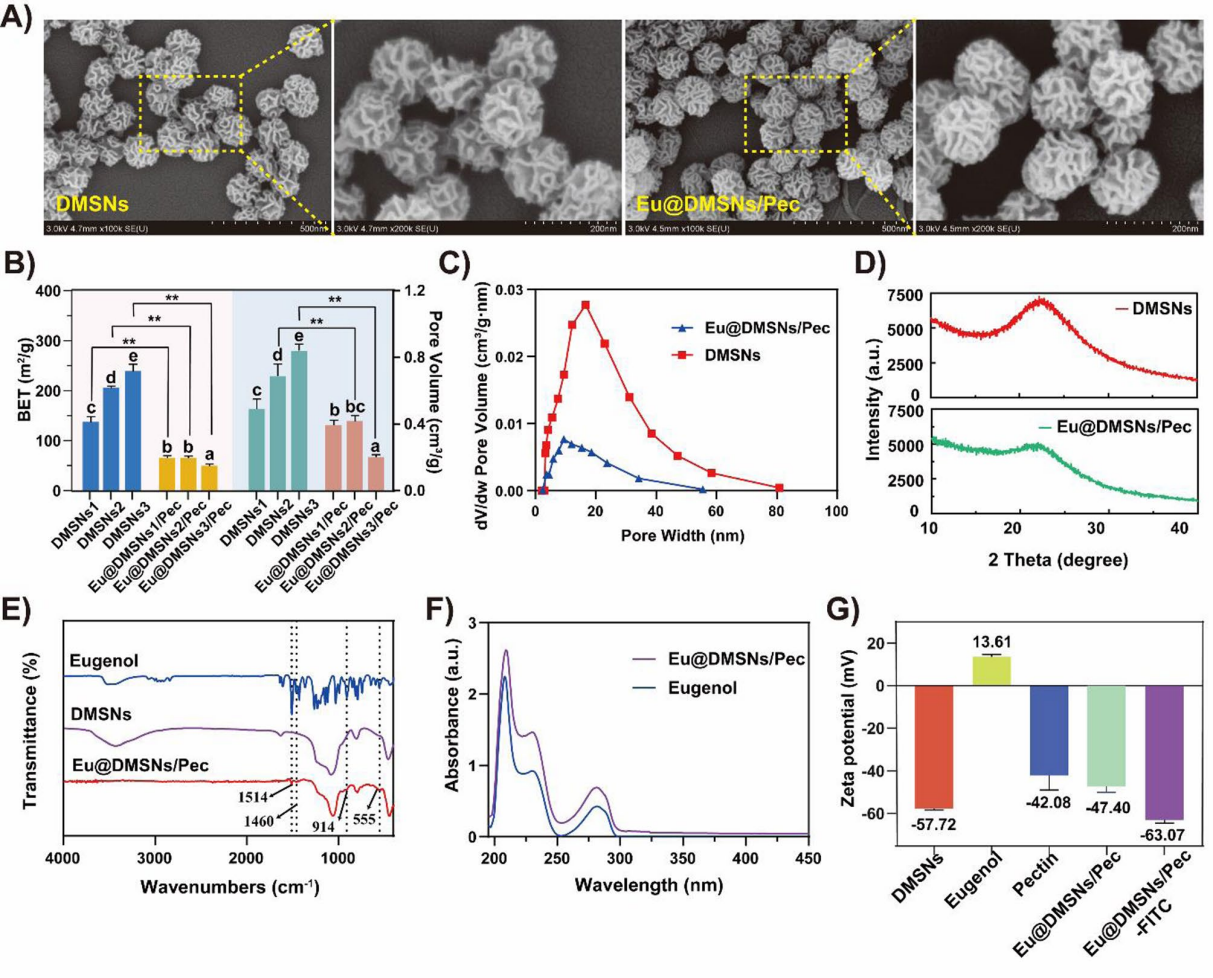
(**A**) SEM of Eugenol and Eu@DMSNs/Pec. (**B**) BET and Pore volume of DMSNs with different sizes before and after biocide loading. (**C**) Pore volume desorption curve of DMSNs and Eu@DMSNs/Pec. (**D**) XRD pattern of DMSNs and Eu@DMSNs/Pec. (**E**) FTIR spectroscopy of DMSNs and Eu@DMSNs/Pec. (**F**) UV–Vis absorption spectrum of DMSNs and Eu@DMSNs/Pec. (**G**) Zeta potential of DMSNs, eugenol, pectin, Eu@DMSNs/Pec and Eu@DMSNs/Pec-FITC

Other characterization results are shown in Fig. 2. After loading eugenol, SEM images showed that the dendritic structure of DMSNs did not change significantly (Fig. 2A). The pore wall was slightly thickened compared with that before loading, and the specific surface area and pore volume decreased significantly. The more biocide loading, the greater the decrease of the two showed a positive correlation trend (Fig. 2B, C). The XRD spectrum demonstrates a shift in diffraction peaks, accompanied by broadened peak deformation and a reduction in peak intensity, likely attributable to the incorporation of eugenol and pectin into the mesopores, resulting in the thickening of the mesoporous walls (Fig. 2D). FTIR spectroscopy (Fig. 2E) showed that Eu@DMSNs/Pec has a stretching vibration absorption peak of C-N bond at 1514 cm^− 1^, which is the result of the formation of amide bond (CO-NH-) between -NH_2_ introduced after grafting on the surface of mesoporous silica and pectin [41]. The absorption peak at 1460 cm^− 1^ is usually related to the bending vibration of methyl or methylene [42], which may be the alkyl side chain in pectin molecules. The absorption peaks at 914 cm^− 1^ and 555 cm^− 1^ are related to the skeleton vibration of the benzene ring in the eugenol molecule [43]. This indicates that pectin is fixed on the surface of DMSNs by amide bond, and eugenol is located in the gap of DMSNs after grafting pectin. The C = C skeleton vibration absorption peak at 1600 cm^− 1^of the aromatic ring in the Eu@DMSNs/Pec-FITC sample (Fig. S1 B)indicates that FITC is successfully cross-linked on the nanocarrier [44]. The UV spectrum (Fig. 2F) showed that Eu@DMSNs/Pec had the same UV absorption peaks as eugenol at 208 nm and 282 nm. The zeta potential results (Fig. 2G) showed that DMSNs were negatively charged with pectin and positively charged with eugenol. The pectin-coated DMSNs and eugenol were loaded by electrostatic adsorption, and the zeta potential of the synthesized Eu@DMSNs/Pec was − 47.40 ± 3.52 mV. After crosslinking FITC, the absolute value of the negative charge of Eu@DMSNs/Pec-FITC is further increased due to the presence of isothiocyanate, which had better dispersion stability [45].

### Responsive release performance of Eu@DMSNs/Pec

**Fig. 3:**
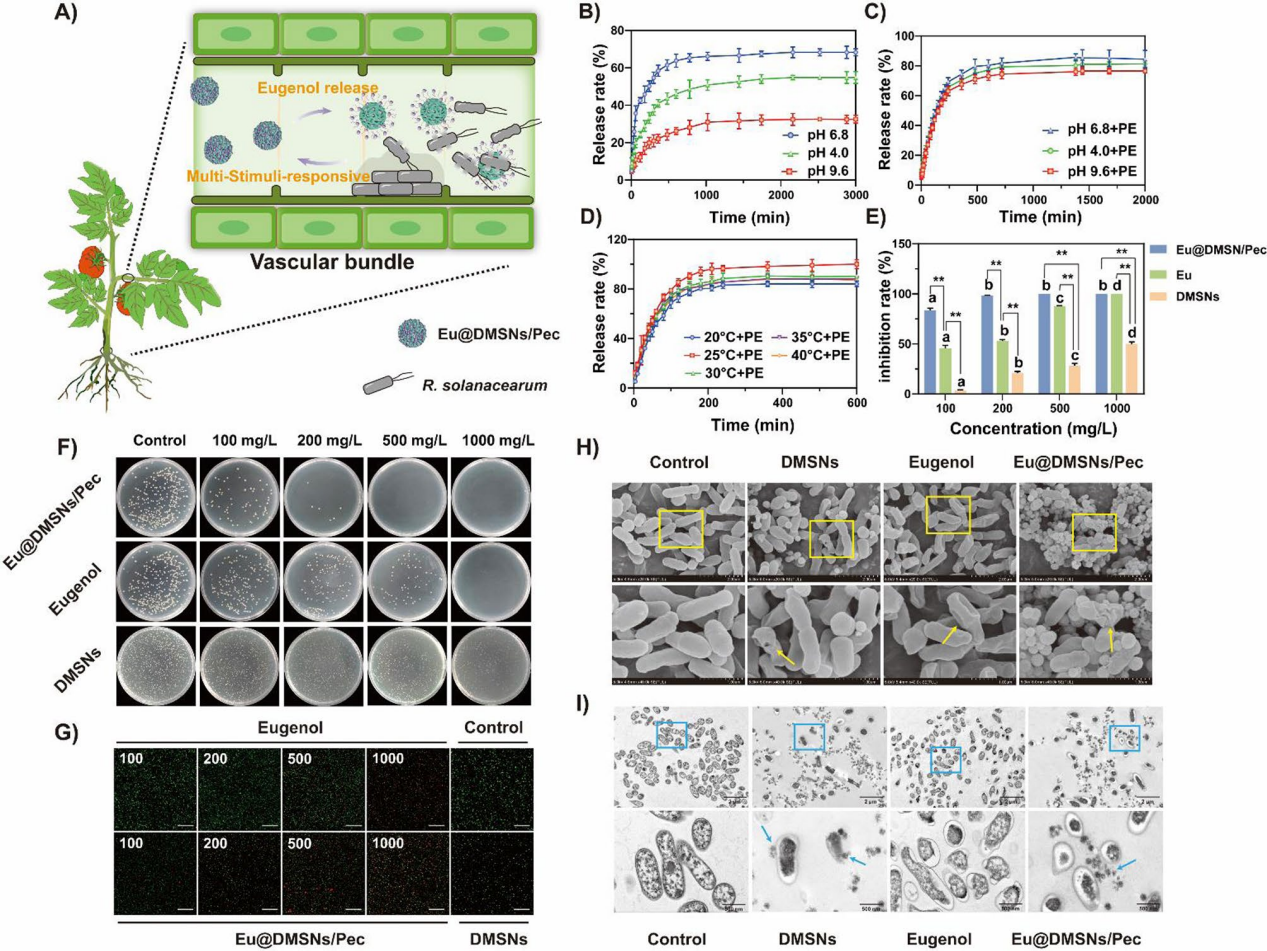
(**A**) Schematic illustration of *R. solanacearum* infection and the Eu@DMSNs/Pec response to their microenvironment. Effect of pH (**B**), pectin (**C**) and temperature (**D**) on the release behaviors of Eugenol from Eu@DMSNs/Pec. (**E**-**F**) In vitro antibacterial activity and photographs of Eugenol, DMSNs and Eu@DMSNs/Pec against *R. solanacearum*. (**G**) Confocal images of *R. solanacearum* after exposure to Eugenol and Eu@DMSNs/Pec for 12 h. (**H**-**I**) SEM and TEM of *R. solanacearum* after exposure to eugenol and Eu@DMSNs/Pec for 12 h

The Eu@DMSNs/Pec system was designed for responsive on-demand release of biocides, specifically targeting the microenvironment of *R. solanacearum* infection (Fig. 3A). According to the literature, *R. solanacearum* can secrete a variety of cell wall degrading enzymes, including polygalacturonase (such as PehA, PehB, PehC) [28]. The role of these enzymes is to decompose pectin, which is a major component of plant cell wall [29]. We introduced pectin as a response factor to regulate the release of eugenol to control the infection of *R. solanacearum*. pH-controlled release curves were depicted by evaluating the responsiveness of nanosystem loaded with eugenol to different pH. As shown in Fig. 3B, the release of eugenol was most effective at a pH of 6.8, with a cumulative release rate of 68.29%±1.54%. At pH 4.0, the release rate was notably lower, and it further decreased to pH 9.6, showing the poorest performance. Overall, the release of eugenol was more effective in acidic conditions, particularly around neutral to slightly acidic pH levels. When the pH is close to neutral, the carboxyl group of pectin molecules is partially deprotonated, and the electrostatic repulsion between pectin molecules increases, resulting in a looser system of mesoporous silicon grafted pectin, which is beneficial to the release of eugenol [46].

In order to understand the release mechanisms and kinetics of eugenol from Eu@DMSNs/Pec, zero-order, first-order, and Ritger - Peppas kinetic models were applied to fit the pH-release data. The fitting results of Fig. S2 and Tab. S2 indicate that the release behavior of Eu@DMSNs/Pec under different pH conditions is more suitable for the first-order kinetic model. The release curve in the presence of pectinase (Fig. 3C) showed that the release trend of different pH was the same as that of previous studies, and the release amount increased to 81.30 ± 1.19% (pH 4.0), 84.45 ± 4.45% (pH 6.8), 76.54 ± 1.22% (pH 9.6), respectively. Under the action of pectinase, the pectin molecules grafted on DMSNs were degraded, resulting in the breakage of the pectin layer on the surface of DMSNs, and the release of eugenol was accelerated [47]. The fitting results of the release kinetics of Fig. S3 and Tab. S3 showed that the release behavior of eugenol in different pH environments in the presence of pectinase was more consistent with the first-order model (R^2^ > 0.95) in the presence of pectinase.

When *R. solanacearum* infects tomato, the temperature of the microenvironment also has a significant effect on the development of the disease [48]. The release behavior of Eu@DMSNs/Pec (20℃, 25℃, 30℃, with pH 6.8,) was investigated under different temperature conditions. The temperature controlled release curve without and with adding pectinase is shown in Fig. 3D and Fig. S4. In general, the cumulative release amount from high to low in the treatment without pectinase was 25℃, 30℃ and 20℃, respectively, and the release behavior was more in line with the first-order model (R^2^ > 0.95) (Tab. S4). The trend after adding pectinase is roughly the same. The cumulative release rate of Eu@DMSNs/Pec was the highest at 25℃, which was 98.00 ± 1.09%, and the lowest at 20℃, which was 84.03 ± 1.28%. Meanwhile, compared with the low temperature environment, the cumulative release rates at 30℃, 35℃ and 40℃ were 90.20 ± 0.63%, 88.18 ± 1.05%, and 87.32 ± 0.99%, respectively. Higher temperature can promote the sustained release of eugenol, which may be related to the low activity of pectinase at low temperature. The release effect is the best at 25℃, which may be due to the fact that the pectin swelling effect on the surface of Eu@DMSNs/Pec is the best at this temperature, which is beneficial to the extension and hydration of pectin molecular chain and promotes the release of eugenol [49]. From the fitting results of the release kinetics of Fig. S5 and Tab. S5, the release behavior of eugenol at different temperatures in the presence of pectinase (pH 6.8) is more in line with the first-order model (R^2^ > 0.95).

### In vitro antibacterial activity

Figure 3E-G illustrates the in vitro antibacterial activity of eugenol and Eu@DMSNs/Pec against R. solanacearum at various eugenol concentrations. Eu@DMSNs/Pec exhibited superior antibacterial efficacy compared to eugenol alone. At 500 mg/L eugenol, Eu@DMSNs/Pec achieved a 100% antibacterial rate, whereas eugenol alone reached 87.79 ± 0.36%. At 100 mg/L and 200 mg/L eugenol, Eu@DMSNs/Pec showed antibacterial rates of 83.7 ± 1.63% and 98.2 ± 0.36%, respectively, significantly higher than eugenol alone (45.71 ± 2.33% and 53.09 ± 0.98%). Additionally, DMSNs alone demonstrated antibacterial activity, with rates of 21.20% at 500 mg/L and 50.03% at 1000 mg/L, indicating that DMSNs possess inherent antibacterial properties and enhance the antibacterial effect of eugenol in Eu@DMSNs/Pec. The results observed by laser confocal microscopy were aligned this trend (Fig. 3G). The *R. solanacearum* in the control group emitted green fluorescence, indicating intact bacterial structure and impermeability to PI. In contrast, the *R. solanacearum* treated with biocides lost activity and emitted intense red fluorescence. The higher the concentration, the stronger the fluorescence intensity, indicating that the cell membrane permeability of the bacteria changed and the *R. solanacearum* was inactivated [32]. A small amount of red fluorescence can be observed in the treatment of DMSNs alone, which proves that DMSNs can help eugenol to change the permeability of bacterial cell membrane so that the Eu@DMSNs/Pec shows better bactericidal effect than eugenol alone.

The morphological structure of the bacteria was observed under SEM and TEM (Fig. 3H, I). DMSNs treatment alone will cause certain physical damage to the bacteria, resulting in cell membrane rupture, and eugenol treatment alone will cause the bacteria to shrink and reduce the intracellular content. Under the treatment of Eu@DMSNs/Pec, relying on the adhesion of DMSNs to pectin carrier, *R. solanacearum* was adhered and enveloped in Eu@DMSNs/Pec (Fig. S6), and the cell damage was severe, significant reduction of intracellular content in bacteria was observed. Eugenol plays a bactericidal role by changing the permeability of bacterial cell membrane and breaking the cell membrane [4]. The silica nanoparticles in the Eu@DMSNs/Pec system can adhere to the surface of the bacteria by forming hydrogen bonds to combine with amino acid residues and then interact with the bacterial cell wall [50, 51]. Pectin is degraded under the action of pectinase released during the infection of *R. solanacearum*, rapidly releasing eugenol, destroying the cell membrane of the bacteria, leading to the leakage of the content material, thereby inhibiting the growth of the bacteria and playing a synergistic role.

### Peroxidation damage of *R. solanacearum*

**Fig. 4:**
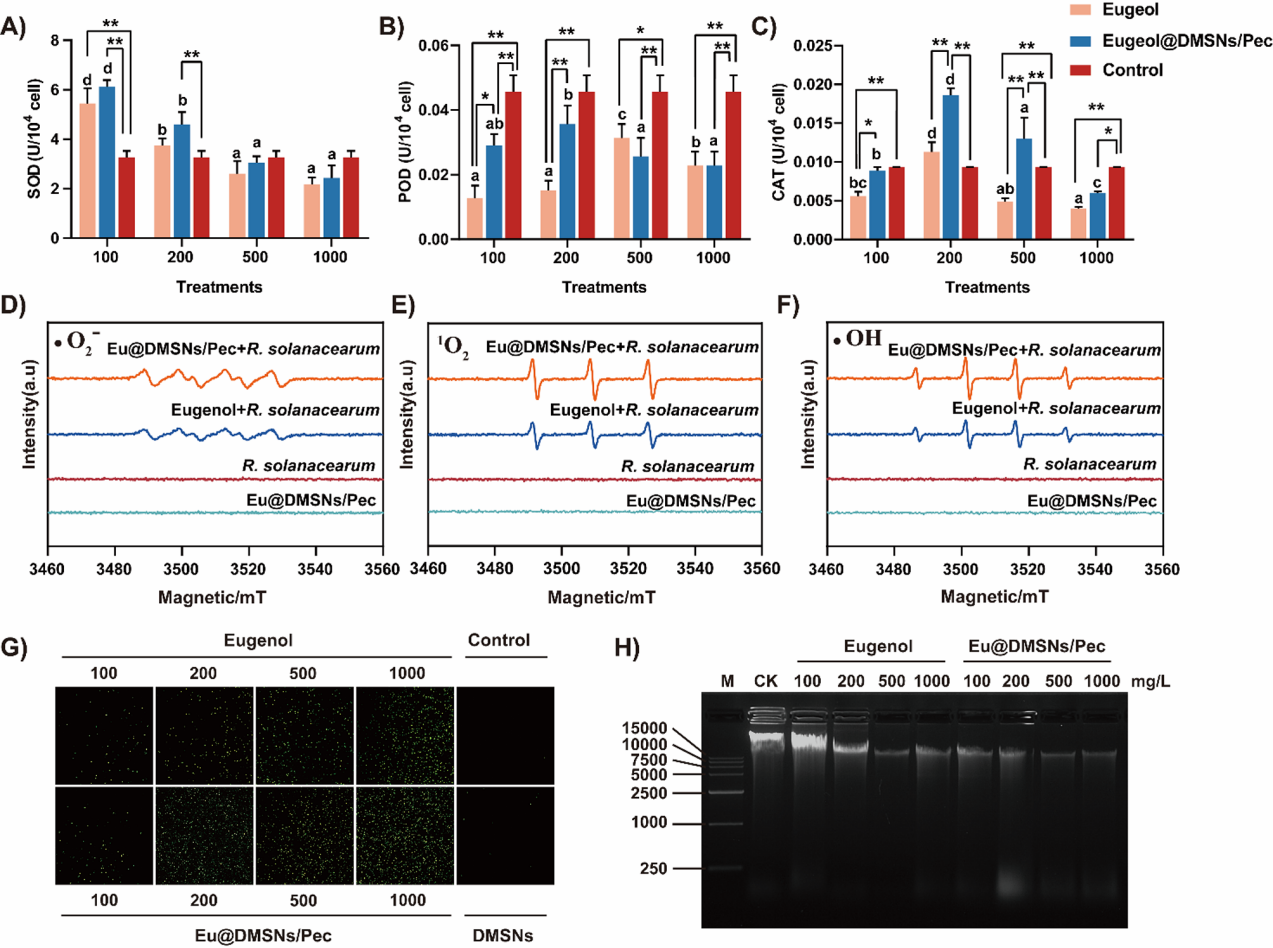
Effects of Eugenol and Eu@DMSNs/Pec on SOD (**A**), POD (**B**) and CAT (**C**) activities in the *R. solanacearum*, respectively. (**D**-**F**) Free radical signal in different treatments identified by EPR. (**G**) Effects of Eugenol and Eu@DMSNs/Pec on the ROS production in the *R. solanacearum.* (**H**) The electrophoresis of extracted *R. solanacearum* genomic DNA (Numbers correspond to concentrations, mg/L, respectively)

Bacteria will initiate a series of stress responses to protect themselves and improve their survival rate when they are exposed to antimicrobial stress. Antibiotics may induce the accumulation of ROS, resulting in oxidative damage. Bacteria remove ROS and repair damage through antioxidant defense system [52]. To further reveal the antibacterial mechanism of Eu@DMSNs/Pec, we measured the activities of SOD, POD and CAT in Eu@DMSNs/Pec and *R. solanacearum* exposed to the same concentration of eugenol. The activity of SOD enzyme increased under the treatment of low concentration of eugenol and Eu@DMSNs/Pec, and decreased after 500 mg/L and lower than that of the control group (Fig. 4A). The activity of POD enzyme was generally lower than that of the control group (Fig. 4B). With the increase of eugenol concentration, the activity of CAT enzyme increased first and then decreased. The activity of CAT enzyme was the strongest when eugenol and Eu@DMSNs/Pec were 200 mg/L. Both lower and higher concentrations showed inhibitory effects (Fig. 4C). EPR detection and ROS fluorescence staining experiments showed that *R. solanacearum* could produce three different types of ROS under the treatment of eugenol and Eu@DMSNs/Pec alone: superoxide radical (•O_2_^−^), singlet oxygen (^1^O_2_) and hydroxyl radical (•OH) (Fig. 4D-F). Compared with eugenol alone, Eu@DMSNs/Pec produced stronger and more ROS signals. This may be because DMSNs alone can also induce *R. solanacearum* to produce a small amount of ROS (Fig. 4G), causing lipid peroxidation to damage the cell membrane. After grafting pectin, it can adsorb and envelope bacteria, thereby limiting the activity and growth of bacteria, which helps Eu@DMSNs/Pec aggravate the damage of eugenol to bacterial cell membranes. Agarose gel electrophoresis results further showed that Eu@DMSNs/Pec treatment reduced the DNA band intensity compared to eugenol alone (Fig. 4H), indicating that Eu@DMSNs/Pec caused more serious DNA damage to bacteria.

### Foliar retention and adhesion

**Fig. 5:**
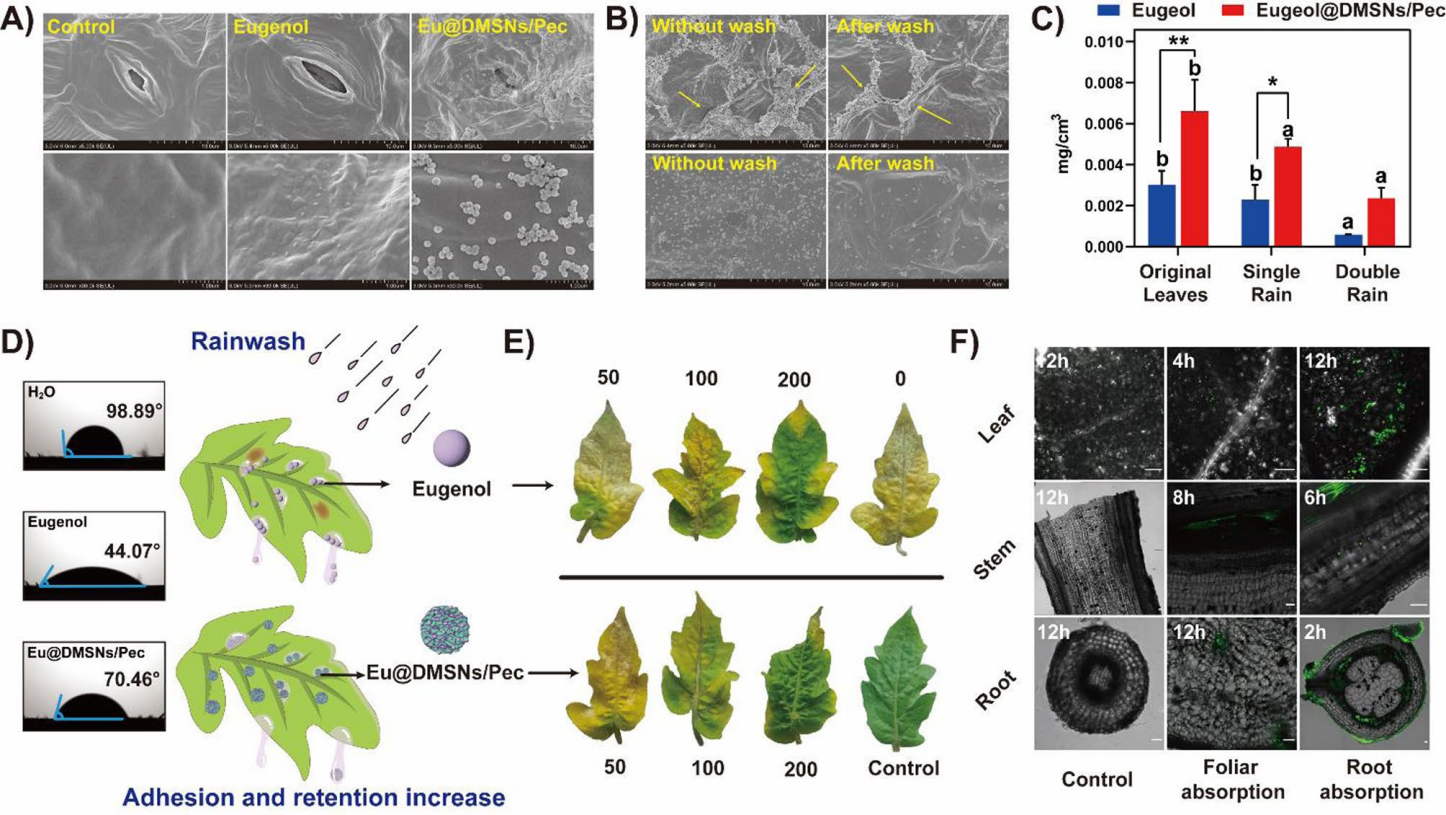
(**A**) Adhesion of eugenol and Eu@DMSNs/Pec to the stomata on the surface of tomato leaves. (**B**) Photographs and (**C**) retention amount of eugenol and Eu@DMSNs/Pec on the surface of tomato leaves after washed. (**D**) Contact angle of eugenol and Eu@DMSNs/Pec and schematic illustration of the foliar retention and adhesion after eugenol and Eu@DMSNs/Pec spraying (**E**) Photographs of eugenol and Eu@DMSNs/Pec in different concentrations against *R. solanacearum* in vitro on tomato leaves. (Numbers correspond to concentrations, mg/L, respectively.) (**F**) Confocal images of different parts of tomato plants (leaves, stems, and roots; scale bar represents 100 μm.) after treatment with Eu@DMSNs/Pec-FITC

In order to evaluate the effect of Eu@DMSNs/Pec in practical application, we studied the retention and adhesion of Eu@DMSNs/Pec on leaves (Fig. 5). SEM images showed that Eu@DMSNs/Pec formed plaques on the leaf surface and gathered into large clumps after spraying Eu@DMSNs/Pec. A large number of Eu@DMSNs/Pec aggregates were observed at the stomata (Fig. 5A). Eu@DMSNs/Pec enhances the interaction with the leaf surface through pectin and other substances. Eugenol may be passively diffused into the leaves through the opening and closing changes of the stomata [47]. The SEM images (Fig. 5B) further showed the adhesion and distribution of Eu@DMSNs/Pec on the surface of tomato leaves before and after water washing. Compared with eugenol treatment alone, Eu@DMSNs/Pec particles containing the same concentration of eugenol showed better residual effect on dried tomato leaves, and the residues were 0.0030 ± 0.00055 mg/cm^2^ and 0.0062 ± 0.0016 mg/cm^2^, respectively (Fig. 5C). A large number of particles adhered to the wrinkles on the surface of tomato leaves. After simulated rainwater washing, many Eu@DMSNs/Pec effectively embedded in the gaps and wrinkles on the surface of tomato leaves. After washing once, the eugenol residue on the surface of tomato leaves in the single eugenol and Eu@DMSNs/Pec treatment groups was 0.0021 ± 0.00035 mg/cm^2^ and 0.0045 ± 0.00061 mg/cm^2^, respectively, which was 64.82 ± 7.73% and 72.19 ± 9.84% of the original adhesion amount. After washing twice, the residual amount was 19.33 ± 0.21% and 37.84 ± 6.69% of the original adhesion amount. Eu@DMSNs/Pec still maintained more eugenol effective adhesion after washing. As a natural polysaccharide with adhesion properties, pectin can enhances the viscosity and adhesion of mixtures by forming composite with other components [49], improving stability. DMSNs/Pec carriers might enhance the adhesion of eugenol on the surface of plant leaves, thereby increasing the retention rate of eugenol on plant leaves and enhancing its efficacy.

The contact angle is a key criterion for evaluating the diffusion and wettability of droplets on the surface of leaves. Figure 5D shows that the contact angle is lower for both eugenol and Eu@DMSNs/Pec than that of water. Specifically, eugenol exhibits a contact angle of 44.07° on tomato leaves, while Eu@DMSNs/Pec shows an even higher contact angle of 70.46°. The surface tension of eugenol was low and unstable, which facilitates its spreading on leaf surfaces and enhances its evaporation. Eu@DMSNs/Pec has a larger contact angle than eugenol alone. However, the leaf surface simulated rain erosion experiments and SEM images of Eu@DMSNs/Pec distribution on leaves indicate that Eu@DMSNs/Pec treatment exhibits better leaf adhesion, thereby enhancing the retention of eugenol on tomato leaves. Its contact angle of 70.46° with the hydrophobic surface of tomato leaves also demonstrates good wettability, allowing eugenol to be slowly released from the nanocarrier rather than rapidly evaporating or degrading like free eugenol, thus improving its durability on plants [53].

### In vivo, in vitro antibacterial activity Eu@DMSNs/Pec

The control effects of Eu@DMSNs/Pec and eugenol on tomato bacterial wilt were evaluated by in vitro leaf infection experiments and in vivo plant infection experiments. As shown in Fig. 5E, S8, normal plant leaves showed a large area of chlorosis and wilting after infection with *R. solanacearum*, and the tip of the leaves showed necrosis. Eugenol and Eu@DMSNs/Pec treatment alleviated the symptoms. The disease index of Eu@DMSNs/Pec at 100 mg/L and 200 mg/L eugenol was significantly lower than that of eugenol treatment. When treated with 100 mg/L eugenol alone, obvious symptoms of *R. solanacearum* infection could be seen in the vascular bundle (leaf vein). The xylem vessels became brown, and the leaves spread outward and lost green, while the vascular bundles in the 100 mg/L treatment group of Eu@DMSNs/Pec were less damaged and the symptoms of dryness were alleviated. Leaf vascular bundle damage was not obvious in 200 mg/L eugenol treatment group, and a small area of chlorosis occurred in plants. In the simulated in vivo experiment, the plants treated with Eu@DMSNs/Pec showed a lower disease index (Tab. S6) during continuous long-term treatment, indicating that it had good continuous protection ability.

Studies have shown that plant silicon (Si) is a quasi-essential nutrient that mediates plant growth and development. Nano-silica can be used as a carrier of micronutrients to deliver micronutrients such as zinc through foliar spraying, which may help to improve the nutritional status and health of tomatoes, thereby indirectly enhancing their resistance to pathogens [54, 55]. The absorption and transport of nano-biocide delivery systems in plants also have an important impact on the use and biological activity of biocides. In order to determine the reasons for the different control effects of Eu@DMSNs/Pec and eugenol alone, the absorption and distribution of Eu@DMSNs/Pec nanoparticles in different parts of tomato plants were studied by leaf spraying and root absorption. The results are shown in Fig. 5F. In general, when nanoparticles enter mesophyll cells through epidermis and stomata, they migrate along the vascular system to other parts for long-distance transport. When nanoparticles act on plant roots, they are absorbed by root hair cells, selectively pass through the cell wall, enter the endodermis from the epidermis, and then transport upward through the xylem pipeline [56]. The particle size of Eu@DMSNs/Pec-FITC was slightly increased compared with that before FITC loading (Fig. S7), but it did not affect its transform in plants. Green fluorescence signals can be observed in tomato leaves, stems and roots treated with Eu@DMSNs/Pec-FITC. Notably, the root absorption treatment observed a shorter time of fluorescence signal transport than the foliar absorption treatment. Its stronger fluorescence signal indicates that Eu@DMSNs/Pec can be more effectively transported to plants through the root absorption pathway. In the root absorption treatment group, obvious fluorescence signals were observed in the stem vascular bundles at 6 h, and obvious fluorescence was also observed within 8 h in the leaf treatment, indicating that Eu@DMSNs/Pec-FITC has the ability to move along the vascular bundles, which can more effectively transport the biocide directly to the infected vascular bundle tissue, increase the local concentration of the biocide and thus enhance the effect of resistance to tomato bacterial wilt.

### Biosafety assessment

**Fig. 6:**
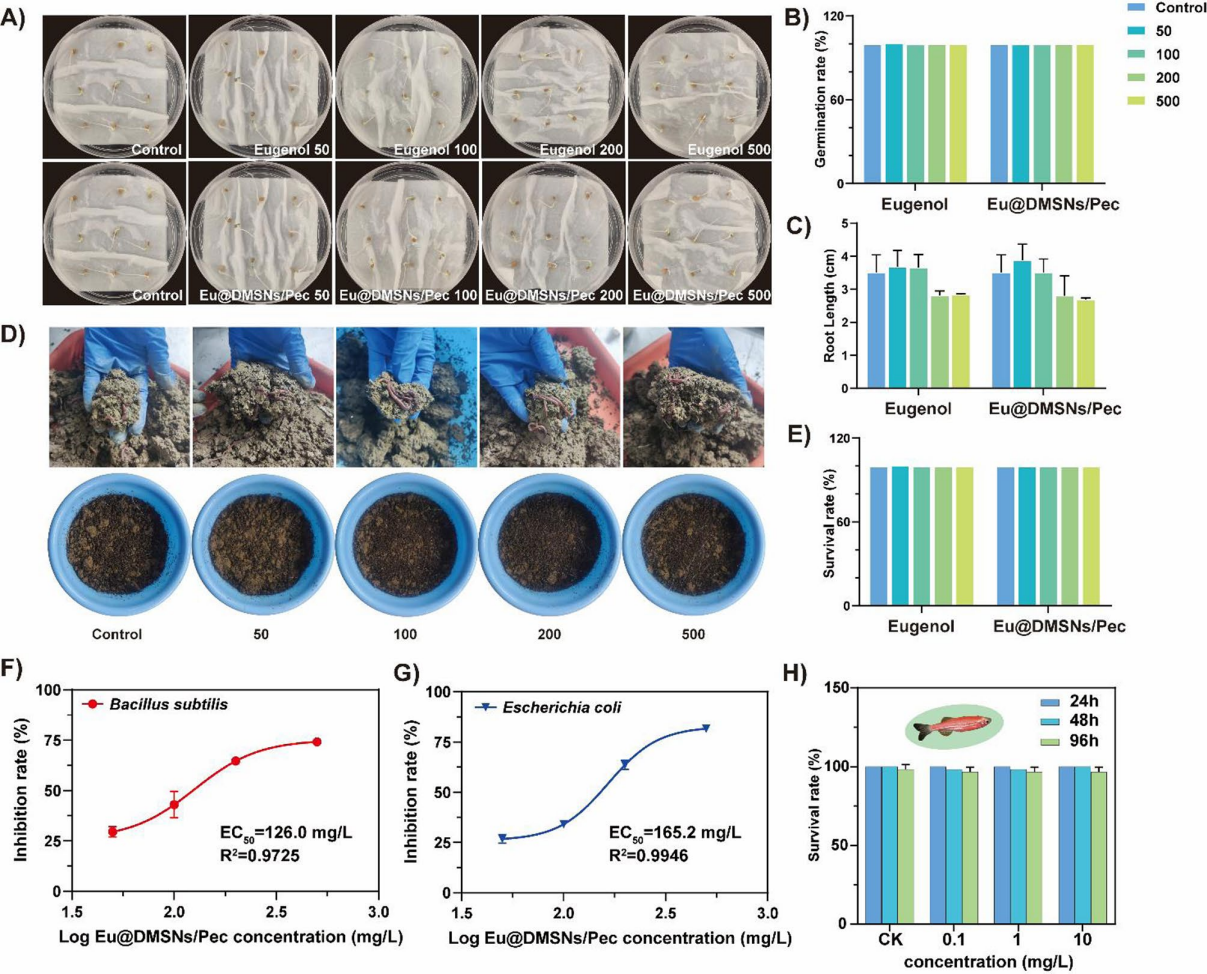
Safety evaluation. (**A**-**B**) Effect of the eugenol and Eu@DMSNs/Pec on the seed germination rate, (**C**) root length, (**D**) earthworm behavior and (**E**) survival. (**F**) Dose-response curve of Eu@DMSNs/Pec against *B. subtilis* and (**G**) *E. coli*. (**H**) Safety evaluation to adult zebrafish

The effects of different concentrations of eugenol and Eu@DMSNs/Pec on tomato seed germination and root growth were studied. As shown in Fig. 6A and B, there was no significant difference in tomato seed germination rate under different treatments, and all seeds could germinate normally. The average root length of the blank control group was 3.51 ± 0.43 cm. Low concentration of eugenol and Eu@DMSNs/Pec treatment could promote the growth of tomato seed root length. The average root length of the 50 mg/L treatment group was 3.68 ± 0.41 cm and 3.89 ± 0.40 cm, respectively. The average root length of the 500 mg/L treatment group was 2.83 ± 0.027 cm and 2.69 ± 0.042 cm, respectively (Fig. 6C). This phenomenon was also found in the studies of other scholars [57]. Studies have shown that eugenol can improve the antioxidant capacity of plants and regulate ion balance, thereby improving the salt tolerance of plants, which may indirectly promote root growth. High concentrations of eugenol may interfere with the plant hormone balance and delay seed germination and root growth [58].

Non-target organisms in the soil can be affected by biocide after entering the soil, so evaluating the toxicity to earthworms in soil is an important means to evaluate the safety of nnanocomposites against their infection [59]. The experimental results are shown in Fig. 6D and E. After 7 days of soil treatment with different concentrations of Eu@DMSNs/Pec, earthworms did not die in the soil. The soil porosity in each treatment was looser than that in the initial treatment, and it had good ventilation and water holding capacity, indicating that Eu@DMSNs/Pec had good safety to the activity and behavior of earthworms. The EC_50_ of Eu@DMSNs/Pec for *B. subtilis* and *E. coli* were 126.0 mg/L and 165.2 mg/L, respectively (Fig. 6F, G). Eu@DMSNs/Pec showed both antibacterial activity against Gram-negative and Gram-positive bacteria, which was consistent with the previous report [60]. Studies have shown that eugenol is an anesthetic suitable for fish and has potential toxicity to the fish’s brain, and the encapsulation of nanoparticles may help reduce its toxicity [61, 62]. In this study, adult zebrafish was used to determine the safety of Eu@DMSNs/Pec to fish. It was found that Eu@DMSNs/Pec had no significant adverse effects on the life activities and development of zebrafish at lower concentrations (0.1, 1 and 10 mg/L) (Fig. 6H). These results are based on short-term (96 h) exposure, and the safety of long-term exposure or higher concentrations needs further evaluation.

## Conclusion

In this study, pectin-modified DMSNs were used as a carrier to successfully construct a microenvironment-responsive eugenol nano-biocide delivery system for *R. solanacearum* infection. The system uses a stable dispersion system formed by grafting pectin on the surface of DMSNs to load eugenol, which increases the load and enhances the selective release ability against the *R. solanacearum* infection micro-environment. The synthesized Eu@DMSNs/Pec system was fully characterized. It was found that the specific surface area, pore size and surface activity of DMSNs were important factors in influencing the biocide loading of eugenol, and the biocide loading capability was up to 72.50%. The release behavior showed that Eu@DMSNs/Pec had significant pH and pectinase stimulation, with varying release amounts under different temperature conditions. Compared with eugenol alone, Eu@DMSNs/Pec has better overall antibacterial effect on *R. solanacearum* and has better resistance to rainwater erosion and foliar retention rate based on the wettability and adhesion of pectin. Eu@DMSNs/Pec-FITC demonstrated efficient transport characteristics in tomato roots, stems and leaves, which enhanced the control effect on tomato bacterial wilt. In addition, Eu@DMSNs/Pec exerts minimal influence on tomato seed germination and root growth, and has low toxicity to non-target organisms such as earthworms. This study demonstrates that the prepared Eu@DMSNs/Pec nanoparticles are an efficient and safe nano-controlled-release formulation.

## Electronic supplementary material

Below is the link to the electronic supplementary material.

Supplementary Material 1

## Data Availability

No datasets were generated or analysed during the current study.

## Abbreviations

DMSNs: Dendritic mesoporous silica
ROS: Reactive oxygen species
Eu: Eugenol
Pec: Pectin
SOD: Superoxide dismutase
CAT: Catalase
POD: Peroxidase
TEA: Triethanolamine
CTAB: Cetyltrimethylammonium bromide
APTES: 3-Aminopropyltriethoxysilane
DMF: Dimethyl formamide
TEM: Transmission electron microscopy
SEM: Scanning electron microscopy
XRD: X-ray diffraction
FTIR: Fourier transform infrared spectroscopy
TGA: Thermogravimetric analysis
EPR: Electron paramagnetic resonance
BET: Brunauer-Emmett-Teller
