# Supplementary material for: Multi-stimuli-responsive pectin-coated dendritic mesoporous silica nanoparticles with Eugenol as a sustained release nanocarrier for the control of tomato bacterial wilt
Source: J Nanobiotechnology. 2025 Mar 8;23:191. doi: 10.1186/s12951-025-03239-8 (PMC11889862; doi:10.1186/s12951-025-03239-8)
Supplement: Supplementary file 1 — Supplementary Material 1 [file 12951_2025_3239_MOESM1_ESM.docx]

**Support information**

**Multi-Stimuli-Responsive Pectin-Coated DMSNs with Eugenol as a Sustained Release Agent for the Control of Tomato Bacterial Wilt**

Xueping Guo^1,#^, Huiyan Li^1,#^, Zhihao Li^1^, Ziqi Cui^1^, Guangming Ma^1^, Aisha Khalfan Nassor^1^, Yi Guan^2,^* and Xiaohong Pan^1,^*

^1^ State Key Laboratory of Agricultural and Forestry Biosecurity & Key Lab of Biopesticide and Chemical Biology, Ministry of Education & Ministerial and Provincial Joint Innovation Centre for Safety Production of Cross-Strait Crops, College of Plant Protection, Fujian Agriculture and Forestry University, Fuzhou, Fujian 350002, P. R. China.

*^2^ Fujian Key Laboratory of Marine Enzyme Engineering, College of Biological Science and Engineering, Fuzhou University, Fuzhou, Fujian, 350116, P.R. China*

^#^These two authors contributed equally

^*^Corresponding Authors

E-mail: panxiaohong@163.com (Xiaohong Pan).

E-mail: gy@fzu.edu.cn (Yi Guan).

**Page No. Content**

4 Preparation of Eu@DMSNs/Pec

5 Characterization of nanoparticles

6 *In vitro* biocide release study

7 Antibacterial experiment and Foliar adhesion and retention

8 Table S1. Disease index for plant leaves. Figure S1. Zeta potential and FTIR of different samples.

9 Figure S2. Fitting curves of (A) Zero-order model, (B) First-order model, (C) Ritger-Peppas model of Eu@DMSN/Pec at varying pH values. Table S2. Release kinetics equations at varying pH values.

10 Figure S3. Fitting curves of (A) Zero-order model, (B) First-order model, (C) Ritger-Peppas model of Eu@DMSN/Pec at different pH values with pectinase. Table S3. Release kinetics equations at varying pH values with pectinase.

11 Figure S4. Fitting curves of (A) Zero-order model, (B) First-order model, (C) Ritger-Peppas model of Eu@DMSN/Pec at varying temperatures. Table S4. Release kinetics equations at varying temperatures.

12 Figure S5. Fitting curves of (A) Zero-order model, (B) First-order model, (C) Ritger-Peppas model of Eu@DMSN/Pec at varying temperatures with pectinase. Table S5. Release kinetics equations at varying temperatures with pectinase.

13 Figure S6: SEM of different treatments in *R. solanacearum* and Table S6. Effect of Eu@DMSN/Pec on *R. solanacearum* (tomato bacterial wilt) in tomato plant.

14 Figure S7: Particle size distribution of Eu@DMSNs/Pec-FITC Figure S8: *in vivo* experiments.

**2. Experimental section**

**2.2 *Preparation of* *Eu@DMSNs/Pec***

200 mg DMSNs was added to 1 M hydrochloric acid (120 mL) to activate the surface hydroxyl group (-OH). The precipitate was collected by centrifugation at 11000 r/min for 10 min after shaking for 20 min. The precipitate was dispersed in 80 mL N, N-dimethylformamide (DMF, 99%, Macklin), and 40 μL 1% 3-aminopropyltrimethoxysilane (APTES, 99%, Macklin) was added. After shaking for 1 h, the precipitate was centrifuged to obtain DMSNs-NH_2_, and 800 mg pectin was dissolved in ultrapure water (200 mL) by magnetic stirring for 40 min until completely dissolved. Then 1-(3-dimethylaminopropyl)-3-ethylcarbodiimide (EDC, 98%, Solarbio) and N-hydroxysuccinimide (NHS, 98%, Aladdin) 20 μL (50 mg/mL, 1:1) were added and the activated pectin solution was obtained by magnetic stirring for 30 min. DMSNs-NH_2_ was dissolved in PBS, and the above pectin solution was slowly added to it. After magnetic stirring for 24 h, the product was centrifuged and washed with ultrapure water to obtain DMSNs/Pec. Then, DMSNs/Pec was dissolved in 40 mL of anhydrous ethanol, and 8 mL (≈8g) eugenol (Eu, 99%, Macklin) dissolved in 40 mL of anhydrous ethanol was slowly added dropwise, and then magnetically stirred for 12 h. After centrifugation at 11000 r/min, the precipitate was collected and freeze-dried to obtain Eu@DMSNs/Pec.

***2.3 Characterization of nanoparticles***

The surface morphology of the samples before and after drug loading was observed by scanning electron microscopy (SEM, Hitachi, SU8010, 5 kV, Japan) at an accelerating voltage of 3.0 kV. Transmission electron microscopy (TEM) images of the samples were collected using a JEM-2100F instrument (JEOL, 200 kV, Japan). Wide-angle X-ray powder diffraction was performed using BRUCKER D8 (Bruker, D8 Focus, Germany). The Zeta potential of the sample was measured on Zetasizer Nano ZS9 (Brookhaven, USA). Fourier transform infrared (FTIR) spectroscopy was performed on a Nicolet IS10 FTIR spectrometer (Thermo Scientific, USA). The UV-visible absorption spectra of the samples (1 μg/mL) were measured by Shimadzu UV-2600 UV-visible spectrophotometer. The pore size distribution and specific surface area of the samples were determined by adsorption analyzer (Micromeritics ASAP 2000, USA). Thermogravimetric analysis (TGA) of the samples was carried out by Seiko S-II (Seiko Corporation, Japan) thermal analyzer in an inert nitrogen atmosphere at a heating rate of 10 °C/min and a temperature range of 25-600 °C. The N_2_ adsorption/desorption isotherm was measured, and the Brunauere Emmette Teller (BET) method was used to calculate the specific surface area of the sample.

***2.4 In vitro biocide release study***

The release process of eugenol from Eu@DMSNs/Pec in different pH (4.0, 6.8 and 9.6), different pH with pectinase (pH 4.0, 6.8 and 9.6) and different temperature with pectinase (pH 6.8, 20 ℃, 25 ℃, 30 ℃, 35 ℃ and 40 ℃) was studied by dialysis method. The release medium was deionized water solution. The sample (10 mg) was dispersed in 10 mL release medium and placed in a dialysis bag. The dialysis bag was sealed and added to the beaker to supplement the release medium to 500 mL. The beaker was placed on a multi-joint magnetic stirrer, and the rotor speed was set to 200 r/min during the release process. 2 mL samples were regularly extracted from the release medium and the same volume of fresh release medium was supplemented. The sample was filtered by a needle filter (0.22 μm) and the eugenol content was detected by high performance liquid chromatography. Chromatographic Elite Hypersil C_18_, conditions were mobile phase: methanol-water (65:35, v/v), flow rate: 1.0 mL/min, detection wavelength: 280 nm, column temperature: room temperature, injection volume: 20 μL. The cumulative release rate was calculated, and the release performance was evaluated. Each experiment was repeated three times.

***2.5 Antibacterial experiment***

The treated bacteria were washed with sterile water and stained with SYTO^®^9 and propidium iodide (PI) dye. After incubation at room temperature in dark for 30 min, the cells were observed under a confocal laser scanning microscope (CLSM). Specifically : An equal volume mixture of SYTO®9 (Life Technologies) and propidium iodide (PI) (6 microliters per 1 mL sample) was freshly prepared, mixed with the sample, incubated at room temperature for 30 min, and then washed three times with sterile water. Subsequently, 20 μL of the stained bacterial suspension was captured between the slide and the 18 mm square cover and examined under a confocal laser scanning microscope (CLSM). The excitation/emission maxima for the SYTO®9 stain and PI were approximately 480/500 nm and 490/635 nm, respectively. Leica Application Suite X was used to analyze the pictures. In addition, SEM and TEM were used to observe the effects of different treatments on the morphological structure and biofilm of *R. solanacearum*.

***2.7 Foliar adhesion and retention***

Eugenol is insoluble in water. In order to achieve uniform dispersion of eugenol in water, we use ultrasonic dispersion equipment (KQ-500DM ultrasonic cleaning instrument) to ultrasonically treat the mixed solution. The conditions of ultrasonic treatment were as follows : ultrasonic power 270 W, ultrasonic frequency 80 kHz, ultrasonic time 30 min. After ultrasonic dispersion treatment, eugenol was uniformly dispersed in aqueous solution to form a stable suspension for comparison with Eu@DMSNs/Pec.

**2.8 *In vitro* antibacterial activity**

**Table S1.** Disease index for plant leaves.

| **Grade** | **Explanation** |
| --- | --- |
| 0 No infection | No lesion on the leaf |
| 1 Weak infection | 5% of leaf area infected |
| 2 Moderate infection | 25% of leaf area infected |
| 3 Significant infection | 50% of leaf area infected |
| 4 Very significant infection | Drying in infected stipes, 75% of leaf area and more infected |

$$Disease index\left( DI \right)=\frac{\sum(Grade\times Number of plants）}{4\times Total number of survey plants}$$

$$Coefficient of variation=\frac{Standard deviation(SD)}{mean value(Mean)}\times100\%$$

**3. Result and discussion**

***3.1 Synthesis and Characterization of Eu@DMSNs/Pec***


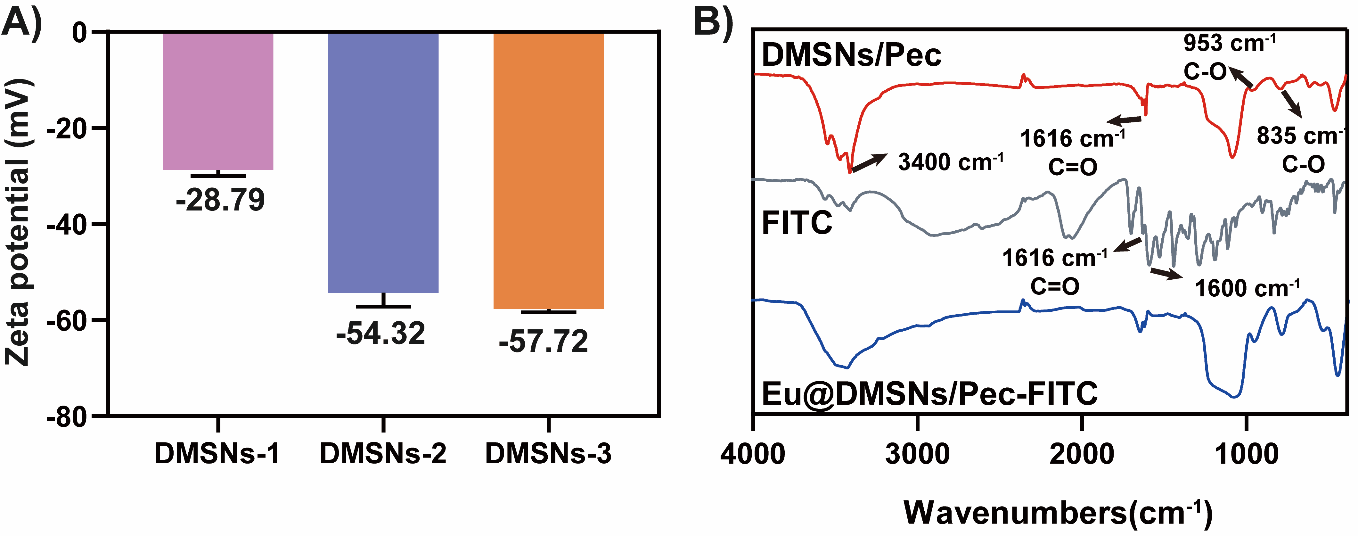


**Figure S1.** Zeta potential and FTIR of different samples.


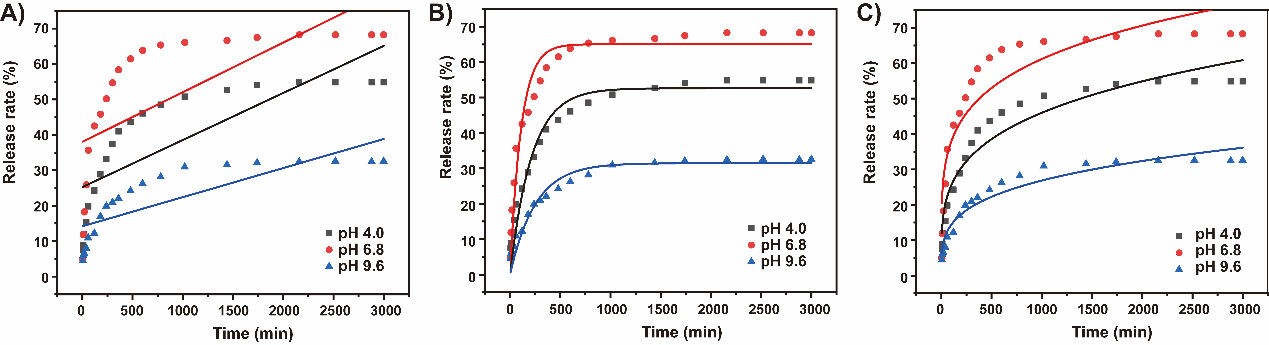


**Figure S2.** Fitting curves of (A) Zero-order model, (B) First-order model, (C) Ritger-Peppas model of Eu@DMSN/Pec at varying pH values.

| **Table S2.** Release kinetics equations at varying pH values. | | | |
| --- | --- | --- | --- |
| Kinetic model | pH | Fitted equation | R^2^ |
| Zero-order | 4.0 | y=0.0133x+25.2552 | 0.6302 |
|  | 6.8 | y=0.0140x+38.0522 | 0.4766 |
|  | 9.6 | y=0.0008x+14.1879 | 0.6628 |
| First-order | 4.0 | y=52.6180(1-e^-0.0047x^) | 0.9469 |
|  | 6.8 | y=65.0619(1-e^-0.0091x^) | 0.9488 |
|  | 9.6 | y=31.4693(1-e^-0.0040x^) | 0.9459 |
| Ritger-Peppas | 4.0 | y = 7.9217x^0.2546^ | 0.9366 |
|  | 6.8 | y = 14.6250x^0.2071^ | 0.8685 |
|  | 9.6 | y = 4.1596x^0.2699^ | 0.9437 |


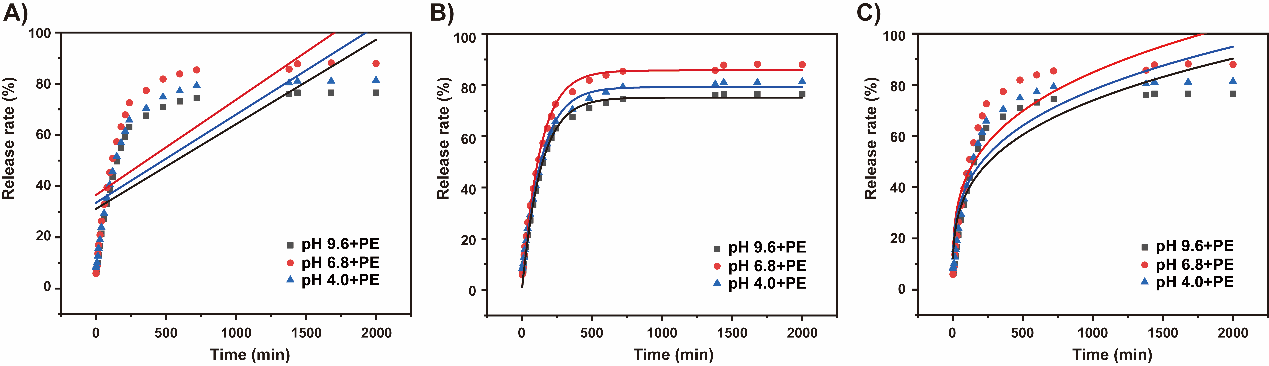


**Figure S3.** Fitting curves of (A) Zero-order model, (B) First-order model, (C) Ritger-Peppas model of Eu@DMSN/Pec at different pH values with pectinase.

| **Table S3.** Release kinetics equations at varying pH values with pectinase. | | | |
| --- | --- | --- | --- |
| Kinetic model | pH | Fitted equation | R^2^ |
| Zero-order | 4.0 | y=0.0331x+31.1058 | 0.5466 |
|  | 6.8 | y=0.0373x+36.4809 | 0.5427 |
|  | 9.6 | y=0.0345x+33.4419 | 0.5648 |
| First-order | 4.0 | y=75.1058(1-e^-0.0074x^) | 0.9944 |
|  | 6.8 | y=85.7264(1-e^-0.0078x^) | 0.9927 |
|  | 9.6 | y=79.2009(1-e^-0.0075x^) | 0.9873 |
| Ritger-Peppas | 4.0 | y = 10.0418x^0.2889^ | 0.8751 |
|  | 6.8 | y = 12.0755x^0.2822^ | 0.8786 |
|  | 9.6 | y = 11.0014x^0.2835^ | 0.8902 |


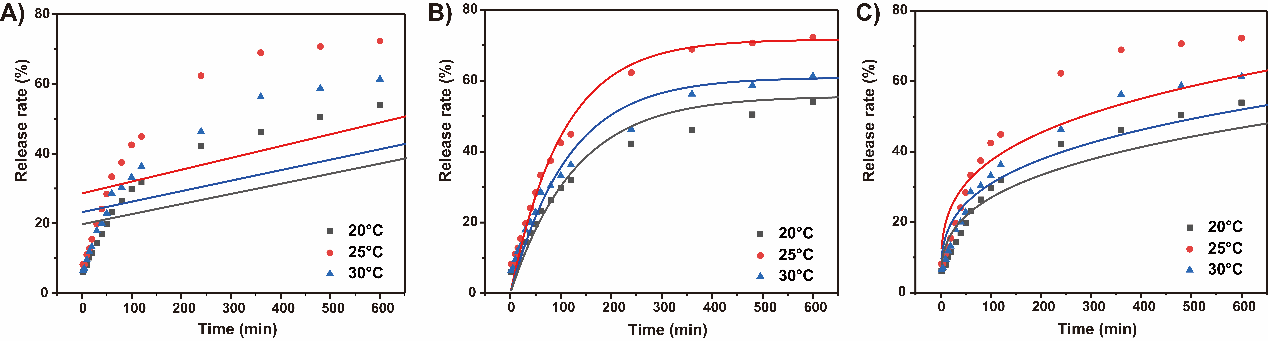


**Figure S4.** Fitting curves of (A) Zero-order model, (B) First-order model, (C) Ritger-Peppas model of Eu@DMSN/Pec at varying temperatures.

| **Table S4.** Release kinetics equations at varying temperatures. | | | |
| --- | --- | --- | --- |
| Kinetic model | Temperatures | Fitted equation | R^2^ |
| Zero-order | 20℃ | y=0.0290x+19.7347 | 0.7244 |
|  | 25℃ | y=0.0339x+28.5772 | 0.5994 |
|  | 30℃ | y=0.0301x+23.1920 | 0.6624 |
| First-order | 20℃ | y=55.6674(1-e^-0.0078x^) | 0.9692 |
|  | 25℃ | y=71.6889(1-e^-0.0096x^) | 0.9882 |
|  | 30℃ | y=60.9441(1-e^-0.0088x^) | 0.9752 |
| Ritger-Peppas | 20℃ | y = 6.4782x^0.3093^ | 0.9491 |
|  | 25℃ | y = 10.4076x^0.2781^ | 0.8877 |
|  | 30℃ | y = 8.1122x^0.2904^ | 0.9227 |


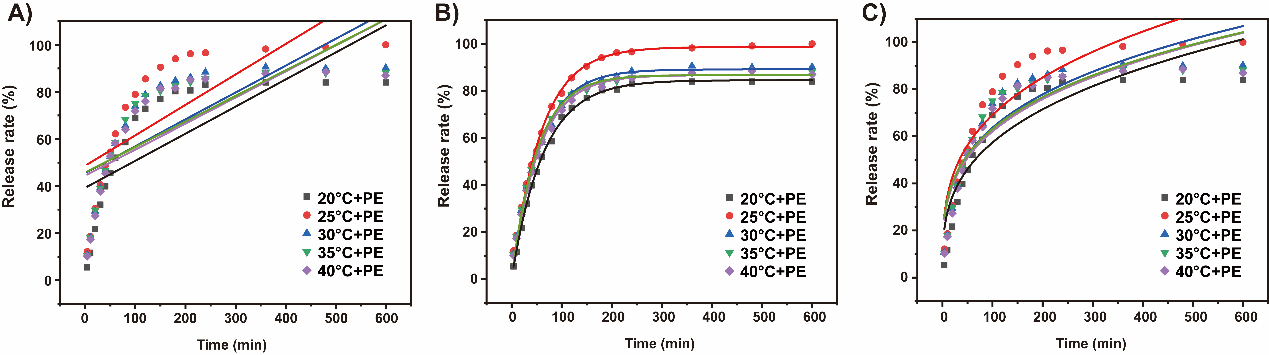


**Figure S5.** Fitting curves of (A) Zero-order model, (B) First-order model, (C) Ritger-Peppas model of Eu@DMSN/Pec at varying temperatures with pectinase.

| **Table S5.** Release kinetics equations at varying temperatures with pectinase. | | | |
| --- | --- | --- | --- |
| Kinetic model | Temperatures | Fitted equation | R^2^ |
| Zero-order | 20℃ | y=0.1150x+39.2374 | 0.5350 |
|  | 25℃ | y=0.1292x+48.6415 | 0.5508 |
|  | 30℃ | y=0.1141x+45.5029 | 0.5397 |
|  | 35℃ | y=0.1084x+45.7661 | 0.5180 |
|  | 40℃ | y=0.1106x+44.4187 | 0.5308 |
| First-order | 20℃ | y=84.5188(1-e^-0.0158x^) | 0.9988 |
|  | 25℃ | y=98.6608(1-e^-0.0170x^) | 0.9959 |
|  | 30℃ | y=89.1379(1-e^-0.0181x^) | 0.9952 |
|  | 35℃ | y=86.7219(1-e^-0.0195x^) | 0.9962 |
|  | 40℃ | y=31.4693(1-e^-0.0040x^) | 0.9959 |
| Ritger-Peppas | 20℃ | y = 7.9217x^0.2546^ | 0.9366 |
|  | 25℃ | y = 17.4489x^0.2988^ | 0.8661 |
|  | 30℃ | y = 16.8375x^0.2888^ | 0.8654 |
|  | 35℃ | y = 17.4187x^0.2797^ | 0.8535 |
|  | 40℃ | y =16.4691x^0.2880^ | 0.8592 |


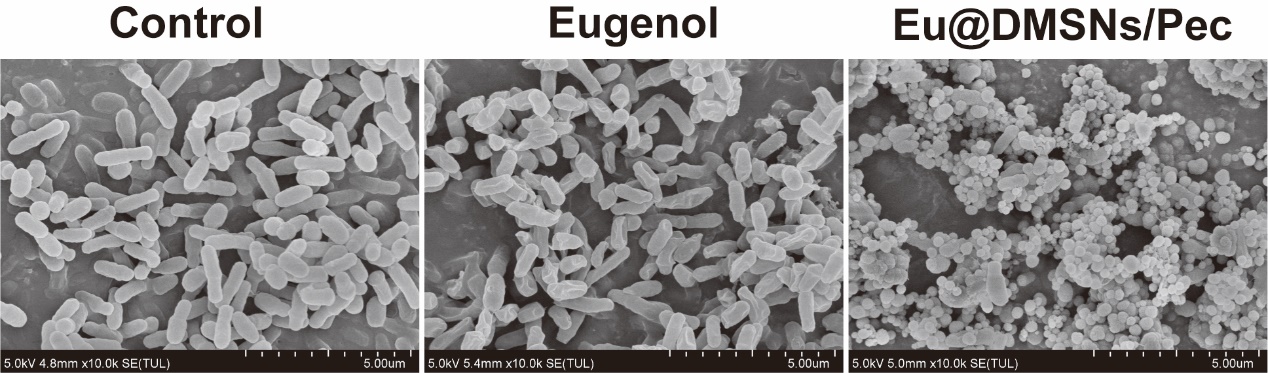


**Figure S6.** SEM of different treatments in *R. solanacearum*

**Table S6.** Effect of Eu@DMSNs/Pec on *R. solanacearum* (tomato bacterial wilt) in tomato plant.

| **Treatment** | **Disease index** | **Standard deviation** | **Coefficient of variation** | **Significant** |
| --- | --- | --- | --- | --- |
| Blank Control | 0 | — | — | — |
| 0 | 4 | — | — | — |
| Eugenol 50 mg/L | 4.80 | 0.42 | 8.78% | Aa |
| Eugenol 100 mg/L | 3.60 | 0.52 | 14.34% | Bb |
| Eugenol 200 mg/L | 2.10 | 0.74 | 35.14% | Cd |
| Eu@DMSNs/Pec 50 mg/L | 4.70 | 0.48 | 10.28% | Aa |
| Eu@DMSNs/Pec 100 mg/L | 3.10 | 0.57 | 18.31% | Bc |
| Eu@DMSNs/Pec 200 mg/L | 1.40 | 0.52 | 36.89% | De |

**Note**: **Lowercase letters** (such as a, b, c) typically represent a significance level of α = 0.05. **Uppercase letters** (such as A, B, C) represent a more stringent significance level of α = 0.01.

**
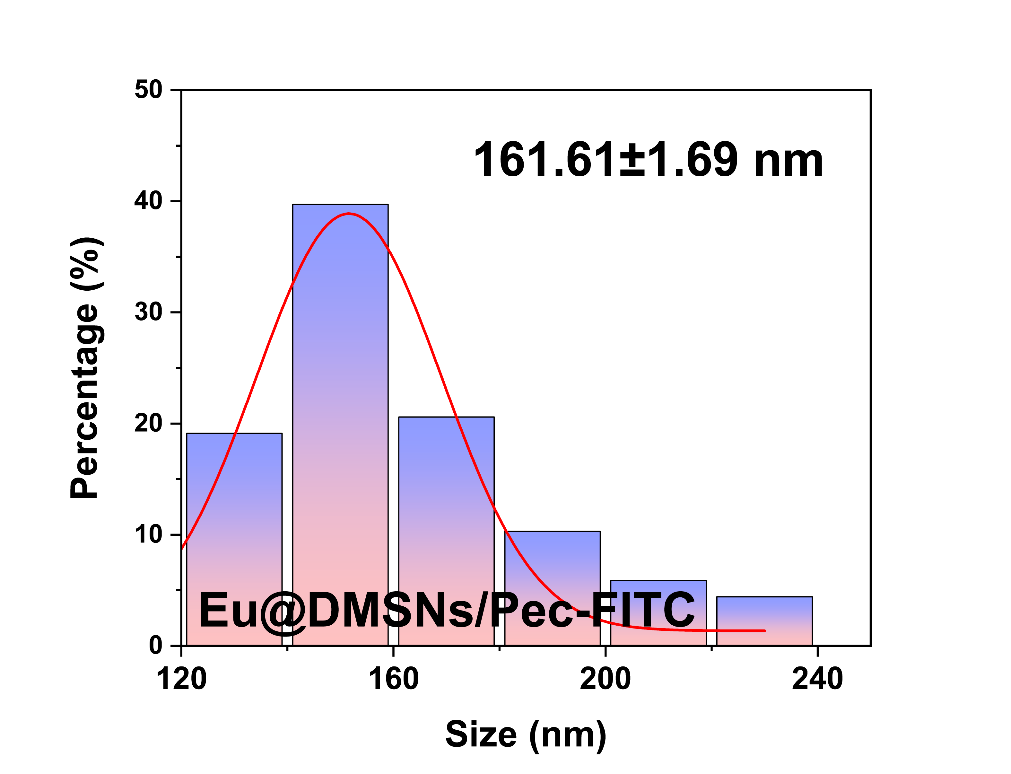
**

**Figure S7:** Particle size distribution of Eu@DMSNs/Pec-FITC.


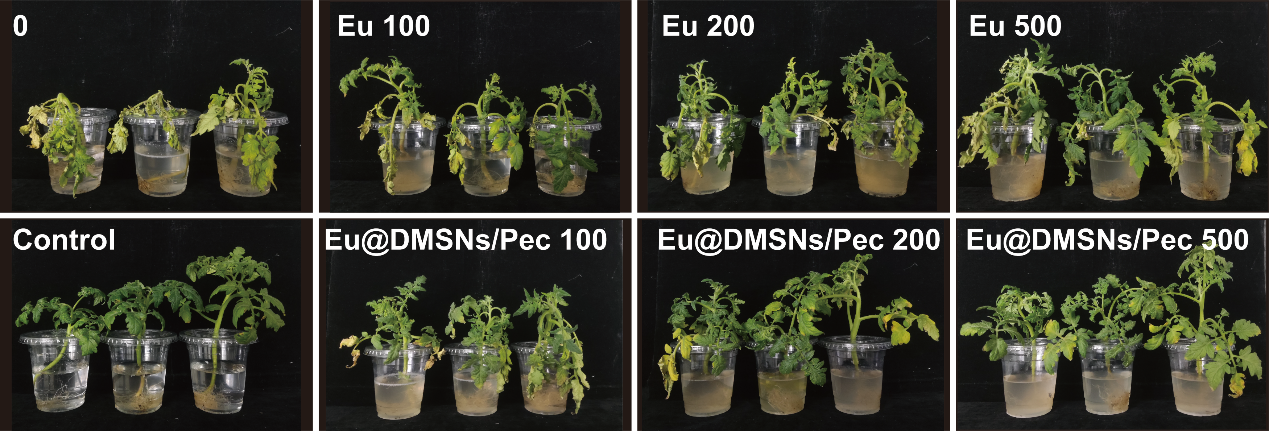


**Figure S8:** *In vivo* plant infection experiments with different treatments.
